# Prevalence of violence by people living with severe mental illness against their relatives and its associated impacts: A systematic review

**DOI:** 10.1111/acps.13516

**Published:** 2023-01-08

**Authors:** Emilie K. Wildman, Deirdre MacManus, Joel Harvey, Elizabeth Kuipers, Juliana Onwumere

**Affiliations:** ^1^ Department of Psychology, King's College London Institute of Psychiatry, Psychology & Neuroscience London UK; ^2^ Department of Forensic and Neurodevelopmental Science King's College London London UK; ^3^ Department of Law and Criminology Royal Holloway, University of London Surrey UK; ^4^ Bethlem Royal Hospital, South London and Maudsley NHS Foundation Trust Beckenham UK

**Keywords:** families, carers, severe mental illness, violence

## Abstract

**Introduction:**

Violence perpetration by adults with severe mental illness (SMI) specifically towards their relatives is a sensitive topic and a largely neglected area that has consequences and implications for different stakeholders, including healthcare providers. This paper sought to systematically review the relevant literature, to identify the types and rates of violence by people with SMI against their relatives, and to develop a detailed understanding of its reported impacts.

**Methods:**

A systematic review, registered with PROSPERO (registration number CRD42019150784), was conducted in accordance with the Preferred Reporting Items for Systematic Reviews and Meta‐Analyses (PRISMA) statement. The review comprised searches of Medline, Embase, PsycInfo and CINAHL databases, supplemented by manual searches. Data from 38 papers using mixed methodologies were reviewed.

**Results:**

Key findings highlighted that relatives experienced different types of violence, including physical, verbal, psychological, financial violence, and violence directed towards property. Different types often co‐occurred. Mothers were the group most likely to report being victims, compared with other relatives. Reported impacts of violence on relatives included mental ill health (e.g., psychological distress, post‐traumatic stress symptoms) and the deterioration, and in some cases the permanent breakdown, of family relationships and the family unit. However, relatives often continued to provide a framework of support for patients, despite risks to their own safety.

**Conclusion:**

Findings speak to the importance of future research extending the focus beyond the identified victimised relative or perpetrator, to also consider the impacts of violence at the family‐wide level, and to improve the outcomes of families exposed to and dealing with violence by individuals living with SMI.

**Summations**

- Definitions, measurement, types, and levels of patient violence varied significantly between the reviewed studies.
- Quantitative and qualitative findings attested to the detrimental impacts of patient violence on mental health and relationship quality in families.
- These impacts often extended beyond the victimised relative and relative‐patient dyad, to the wider family network, warranting greater consideration of the family as an integrated unit in future research and practice.

**Limitations**

- Only peer‐reviewed and English language published papers were included; potentially relevant, non‐English language, and/or unpublished literature were not examined.
- Several methodological issues, common across the reviewed studies (e.g., recruitment and sample size), raise questions about selection and non‐response bias, sample representativeness, and applicability of findings beyond populations studied.

## 1 INTRODUCTION

Globally, an estimated 259.1 million people are in informal (unpaid) caregiving roles,^1^ supporting people with a wide range of conditions, including those living with severe mental illnesses (SMI).^2^, ^3^, ^4^ Informal carers can be unrelated, but are mostly close relatives (e.g., parents).^5^ We therefore use the term ‘relatives’.

While relatives play an invaluable role in supporting patients with SMI, detrimental impacts on their own health are well‐documented.^6^, ^7^, ^8^ Impacts include carer burden, including feelings of shame, loss, and self‐blame^9^, ^10^; common mental disorders^11^, ^12^; burnout^7^, ^13^; and social isolation.^12^ They can also be the targets of patient perpetrated violence,^14^ which constitutes a particular burden for relatives,^15^ yet remains largely overlooked^4^, ^16^ particularly in terms of the lived experience and impact.^17^

Data from a systematic review, exploring impacts of patient violence on relatives, highlighted burden, fear, helplessness, and trauma.^18^ However, the review's focus on psychosis‐only populations, precluded wider consideration of issues relevant to those with other SMI diagnoses. Furthermore, a single focus on physical violence meant that other types (e.g., verbal, psychological, sexual, financial) went unexamined. This is an important limitation since different types of patient violence towards relatives often co‐occur.^19^, ^20^ A recent literature review on violence against relatives by people with SMI, which also adopted a single focus on physical violence, found that most studies reported past year victimisation rates of 20% or higher.^21^ However, searches were restricted to one electronic database and data published between 2015 and 2019, limiting the scope of the findings.

To date, there are no systematic reviews describing the rates and types of violence by adults with SMI against their relatives, and synthesising evidence on the impacts of violence for the relative, and wider family.

### 1.1 Study aims

To systematically review published literature to address two research questions: (i) what are the types and prevalence rates of violence perpetrated by adults with SMI, against their relatives? (ii) what are the impacts of patient violence for relatives?

## 2 MATERIAL AND METHODS

### 2.1 Design

A systematic review, conducted in accordance with the Preferred Reporting Items for Systematic Reviews and Meta‐Analyses (PRISMA) statement,^22^ of peer reviewed manuscripts synthesising both quantitative and qualitative findings. The protocol was registered with PROSPERO (registration number CRD42019150784).

### 2.2 Search strategy

A combination of key text‐words, phrases, and medical subject headings were employed, with truncations, wild cards, and focus/explode functionality. Selected terms and headings were tailored to each database (supplementary material). No restrictions were placed on the time period of searches; four database searches (Medline, PsycInfo, Embase, CINAHL) were undertaken from database inception to 23rd October 2019. Searches were supplemented by forward and backward citation tracking of included studies, and relevant systematic reviews. Database searches were updated on 2nd February 2022 using publication date limits. No additionally relevant literature was identified.

### 2.3 Inclusion/exclusion criteria

#### 2.3.1 Terminology

##### Violence

Violence was operationalised broadly to encompass physical, sexual, verbal, emotional, psychological, and financial violence towards another person and/or violence towards property. This operationalisation reflects the United Nations' definition of domestic violence,^23^ and individual regions (e.g., UK).^24^

##### Severe mental illness

Severe mental illnesses was defined as comprising: schizophrenia spectrum disorders (SSDs) (and related psychotic disorders), bipolar affective disorder (BPAD), and major depressive disorder (MDD). This accords with the literature,^3^ and the diagnostic and statistical manual of mental disorders (DSM‐5).^2^

This review included English language, peer reviewed literature. It was limited to: quantitative studies reporting rates of patient violence towards relatives; quantitative, qualitative and/or mixed methods studies reporting impacts of patient violence. Eligible papers included patient samples aged ≥16, with a SMI diagnosis and reported violence perpetration against a relative (and/or sampled relatives reporting violent victimisation from a family member with SMI). Excluded papers: (i) reported only on: general population samples, patient samples without formal diagnosis, and/or individuals with organic mental (e.g., dementia) and neurodevelopmental (e.g., autism spectrum) disorders; (ii) were mixed diagnostic samples with <75% patients diagnosed with a SMI; (iii) comprised samples aged <16 years and/or comprised mixed ages, but <50% were aged >16 years^(^ * ^)^; (iv) where violence prevalence rates could not be derived (i.e., as a result of unclear reporting); and (v) reported only on: familial homicide; patient victimisation; intimate partner violence (IPV)^(^ † ^)^.

### 2.4 Article selection and extraction

After duplicate removal, citation titles and abstracts were screened in Endnote by the first author, against eligibility criteria. A second reviewer independently screened a randomly selected 10% of citations, with high agreement (*κ* = 0.90). Disagreements were resolved through discussion. Full texts of remaining papers, and those identified through manual searches, were assessed by the first author. A second reviewer independently screened full texts of a randomly selected 10% of the included studies, with 100% agreement.

### 2.5 Data synthesis

Study population (sample sizes; demographics), country, design and clinical setting, patient‐relative relationship type were extracted from all included studies, and tabulated.

#### Quantitative

Types of violence measured; time periods assessed; prevalence rates, including details about violence by relationship type and SMI diagnosis (where reported); impacts of violence (where reported) were synthesised and tabulated from quantitative and mixed methods studies.

#### Qualitative

A thematic synthesis was conducted. In accordance with Thomas and Harden,^25^ codes were generated and data (e.g., participants' quotes; authors' interpretations) were extracted. Descriptive themes were constructed by organising codes based on related concepts. Analytical themes were generated through interrogation of descriptive themes, in light of the review's focus on experiences and impacts of patient violence. The synthesis was led by the first author. Coding and thematic categories were regularly discussed with the research team, to ensure consensus.

### 2.6 Study quality

The Hawker tool^26^ was used to appraise the quality of qualitative and mixed methods studies. Quantitative studies were assessed using the AXIS tool.^27^ Quality appraisal was conducted by the first author. Two additional reviewers independently appraised a randomly selected 20% of the included studies with high agreement. Disagreements were resolved through discussion.

## 3 RESULTS

**FIGURE 1:**
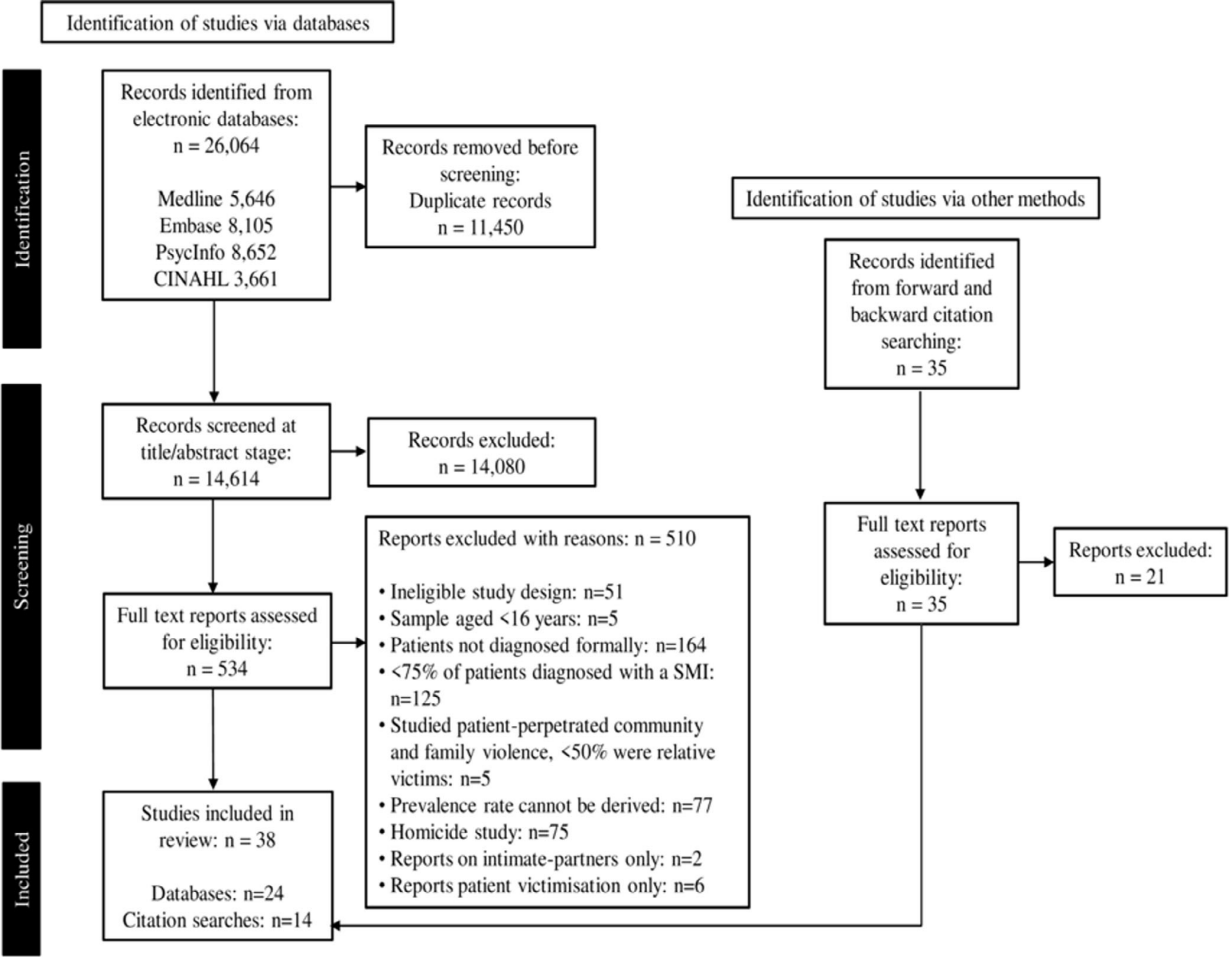
PRISMA flow diagram of study identification.

Database searches identified 26,064 articles, reducing to 14,614 following duplicate removal. Total of 24 articles met full criteria for selection and manual searches identified a further 14 relevant articles. In 38 studies employing quantitative (*n* = 26), qualitative (*n* = 11), and mixed methodologies (*n* = 1) were included. Though searches were completed from database inception, publication dates for reviewed papers fell between 1997 and 2020. The article selection process is outlined in Figure 1.

### 3.1 Study characteristics

Study characteristics are summarised in Table S1 (supplementary material). All studies employed a cross‐sectional design, except one, which used a cohort design.^28^ Most studies (*n* = 26) focused on community‐dwelling patients, seven comprised inpatient samples,^4^, ^28^, ^29^, ^30^, ^31^, ^32^, ^33^ and five were mixed inpatient and community samples.^14^, ^34^, ^35^, ^36^, ^37^

### 3.2 Study quality

Quality appraisal of quantitative studies (supplementary material) identified common issues. For example, selection bias, non‐response and possible confounding variables were not fully considered and/or addressed within most studies. Quality assessment of qualitative and mixed methods studies identified similar issues (supplementary material). Discussion of ethical considerations, researchers' reflexivity and potential bias were also often absent.

#### 3.2.1 Participant samples

Samples comprised: relatives only (*n* = 27)^(^ ‡ ^)^; patients only (*n* = 2)^28^, ^43^; and both relatives and patients (*n* = 6).^14^, ^30^, ^31^, ^33^, ^44^, ^45^

##### Relatives

There were 4708 relative participants. Gender was reported for 3913 relatives; females represented 74% (*n* = 2885). In 12 studies described sample ethnicities, predominantly comprising participants classified as white. Where reported, participant age ranged from 20 to 90 years. Parents of patients comprised the largest relative group, accounting for 73% (*n* = 3440), followed by: 11% (*n* = 501) siblings; 9% (*n* = 408) spouses/partners; 4% (*n* = 189) adult children; and 2% (*n* = 78) other relatives.

##### Patients/perpetrators

While many studies did not directly sample patients, their demographic details were reported. In total, 5045 patients were recorded. Gender was reported for 4605 patients; males accounted for 63% (*n* = 2902). Nine studies reported sample ethnicities, mostly comprising participants classified as white. Where reported, patient age ranged from 8 to 65 years. Eight studies sampled patients (*n* = 700); 59% (*n* = 415) were male. In 25 studies, 100% of the patient sample had schizophrenia spectrum and related psychotic disorders diagnoses. The remaining studies reported on mixed diagnostic patient samples. Among patients (*n* = 5045), reported SMI diagnoses included: schizophrenia spectrum and related psychotic disorders (77%, *n* = 3902); BPAD (13%, *n* = 630); and MDD (3%, *n* = 151).

#### 3.2.2 Quantitative assessment of violence

Table 1 details types of violence studied, assessment measures, and violence rates (including, where reported, rates by: violence type, diagnosis, and relative‐patient relation). All quantitative (*n* = 26) and mixed methods (*n* = 1) studies used self‐report methods to study violence. Seventeen studies used existing or modified versions of self‐report tools, and 10 employed questions derived by the researchers. Assessment time frames varied, ranging from 2‐weeks to lifetime; some included multiple assessment periods. Operationalisations of violence varied, though all studies examined physical violence. While five studies examined physical violence only, most examined additional types. These included: threats (*n* = 5), and threats involving a weapon (*n* = 2); property destruction (*n* = 8); verbal (*n* = 9); psychological (*n* = 7); financial (*n* = 3), and sexual violence (*n* = 1).

**TABLE 1:** Prevalence rates of patient violence: Key findings from quantitative and mixed methods studies.^a^

Author, year	Sample (*N*)	Violence measurement	Types of violence in definition	Prevalence of violence by type where reported %		Key findings regarding prevalence rates of patient violence against relatives			
						Relationship			Patient SMI Dx (source of Dx)
**Studies examining patient‐reported violence perpetration (*N* = 2):**									
Fawzi et al., 2013^43^	150	Abused Parent Questionnaire^46^		2‐month prevalence		100% parent sample. Perpetration prevalence: Against mothers: 92% Against fathers: 23%			100% Psychosis (confirmed using DSM‐IV). No significant differences in violence perpetration by Dx.
				Mothers		Fathers			
			Physical &financial	72		32.8			
			Verbal	33		18			
			Psychological	9.7		11.5			
Elbogen et al., 2005^28^	245	National Institute of Mental Health Diagnostic Interview Schedule (adapted)^47^, ^48^		12‐month prevalence		Details about relationship type NR.			Mixed Dx sample (clinical records). No significant differences in violence perpetration by Dx.
			Physical Threat (weapon)	19.2					
**Studies examining relative/carer‐reported violent victimisation (*N* = 25):**									
Lauber et al., 2003^49^	64	Interview for Measuring Burden on Family (IMBF)^50^		2‐week prevalence		Mixed relation sample. Further details NR.			100% SZ (clinical records).
			Physical	38					
			Property	45					
			Threat	58					
^b^Madathumkovilakath et al., 2018^51^	270	Overt Aggression Scale (OAS adapted)^52^		1‐month prevalence		Mixed relation sample. Further details NR.			Mixed Dx sample (confirmed using ICD‐10). Further details NR.
			Physical	25.6					
			Property	67					
			Verbal	95.6					
^b^Varghese et al., 2016^37^	100	OAS (adapted)^52^; Aggressive Behaviour & Intervention Checklist^37^		1‐month prevalence		Mixed relation sample. Further details NR.			Mixed Dx sample (confirmed using ICD‐10). Further details NR.
			Property	75					
			Physical	80					
			Verbal	95					
Labrum, 2017^53^	243	MacArthur Community Violence Instrument (MCVI adapted)^54^; psychological & financial (study‐specific)		6‐month prevalence		Mixed relation sample. AOR indicate lower odds of victimisation in parents.			Mixed Dx sample (relative confirmed formal Dx). Relatives' VV did not differ significantly by patient Dx.
			Physical	15					
			Financial	19					
			Psychological	41					
Labrum & Solomon, 2016^40^	573	MCVI (adapted)^54^		6‐month prevalence		Mixed relation sample. AOR indicate lower odds of victimisation in parents.			Mixed Dx sample (relative confirmed formal Dx). No significant differences in VV by patient Dx.
			Physical Threat	21					
Labrum et al., 2015^55^	217	MCVI (adapted)^54^; psychological & financial (study‐specific)		6‐month prevalence		Mixed relation sample. Further details NR.			Mixed Dx sample (relative confirmed formal Dx). Further details NR.
			Physical	15					
			Financial	20					
			Psychological	42					
Labrum & Solomon, 2017^41^	573	MCVI (adapted)^54^	Physical	6‐month	Since Dx	Mixed relation sample. No significant differences in victimisation by relationship type.			Mixed Dx sample (relative confirmed formal Dx). Further details NR.
			Any	22	47				
			Minor	14	27				
			Severe	8	20				
Labrum & Solomon, 2016^42^	573	MCVI (adapted)^54^		6‐month	Since Dx	Mixed relation sample. Further details NR.			Mixed Dx sample (relative confirmed formal Dx). Further details NR.
			Threat (weapon)	4.2	10				
			Physical (weapon)	2.3	4.5				
Kageyama et al., 2016^56^	400	MCVI (adapted)^54^	Physical	12‐month prevalence		100% parent sample. Victimisation prevalence: Mothers: 35% Fathers: 32%			100% SZ (relative confirmed formal Dx).
			Minor	34					
			Severe	8.5					
Kageyama & Solomon, 2018^57^	289	9‐item checklist (study‐specific)		12‐month prevalence		100% parent sample. Further details NR.			100% SZ (relative confirmed formal Dx).
			Physical	36					
Kageyama et al., 2016^58^	379	14‐item checklist (study‐specific)		12‐month prevalence		100% parent sample. Further details NR.			100% SZ (relative confirmed formal Dx).
			Physical	34.8					
			Psychological	58.1					
Loughland et al., 2009^59^	106	Perceptions of Prevalence of Aggression Scale^60^		12‐month prevalence		Mixed relation sample. No differences in relationship type between violence subgroups			100% SSD (clinical records) Further details NR.
			Physical Verbal	None‐mild	Moderate‐severe				
			Sexual	22.6	77.4				
Wang et al., 2019^4^	208	Study‐specific questions		12‐month prevalence		Mixed relation sample. Victimisation prevalence: Parents: 76% Children: 76% Spouse: 71% Sibling: 66%			Mixed Dx sample (relative confirmed formal Dx). No significant differences in VV by patient Dx.
			Property	38					
			Physical	45.2					
			Threat	54.3					
			Verbal	61.5					
Vaddadi et al., 2002^45^	101	Burden on Family Interview Schedule (BFI adapted)^61^		12‐month	Since IO	Mixed relation sample. Further details NR.			Mixed Dx sample (confirmed using DSM‐IV). Further details NR.
			Threat	22	40				
			Property	22	41				
			Physical	24	40				
			Verbal	42	64				
Chan, 2008^20^	61	Conflict Tactics Scale‐Revised^62^	Physical	12‐month	Lifetime	Mixed relation sample. Further details NR.			100% SZ (relative confirmed formal Dx).
			Minor | Severe Psychological	31 26	52 38				
			Minor | Severe	64 44	80 64				
Kageyama et al., 2015^63^	301	5‐item checklist (study‐specific)		12‐month	Lifetime	Mixed relation sample. Victimisation prevalence:			100% SZ (relative confirmed formal Dx).
			Physical	27.2	60.9	12‐month		Lifetime	
						Mother	24%	51%	
						Father	17%	47%	
						Spouse	11%	24%	
						Sister	10%	24%	
						Brother	5%	18%	
Ferriter & Hubband, 2003^29^	26	Interview & Behavioural Problem Checklist (adapted)^64^		Prevalence since IO		100% parent sample. Further details NR.			100% SZ (clinical records).
			Physical	65					
			Verbal	69					
Hanzawa et al., 2013^34^	116	Study‐specific questions		Prevalence since IO		Mixed relation sample. Victimisation prevalence:			100% SZ (confirmed by psychiatrist).
			Physical	48.3		Mother: 51%			Son: 83%
			Verbal			Father: 50%			Daughter: 50%
						Brother: 56%			Husband: 33%
						Sister: 38%			Wife: 50%
Vaddadi et al., 1997^33^	101	BFI (adapted)^61^		Prevalence since IO		Mixed relation sample. Further details NR.			Mixed Dx sample (confirmed using DSM‐IV). VV significantly greater in SZ than MDD relatives. No differences between SZ and BPAD.
			Property	16					
			Physical	32					
			Threat	46					
			Verbal	61					
Kageyama & Solomon, 2018^65^	353	MCVI (adapted)^54^; Psychological (study‐specific)	Physical	Lifetime prevalence		100% parent sample. Further details NR.			100% SZ (relative confirmed Dx).
			Minor	74.2					
			Severe	28.4					
			Psychological	56.1					
Kageyama & Solomon, 2019^66^	113	MCVI (adapted)^54^	Physical	Lifetime prevalence 46		100% sibling sample. No significant differences in victimisation.			100% SZ (relative confirmed Dx).
Kageyama, Solomon, et al., 2018^67^	277	14‐item checklist (study‐specific)		Lifetime prevalence		Mixed relation sample. Further details NR.			100% SZ (relative confirmed Dx).
			Physical	75.8					
			Psychological	87.7					
Onwumere et al., 2014^14^	72	Camberwell Family Interview (CFI)^68^		Lifetime prevalence		Mixed relation sample. Further details NR.			100% Psychosis (clinical records).
			Property	9					
			Physical	24					
Smith & Greenberg, 2008^69^	136	Study‐specific questions		Lifetime prevalence		100% sibling sample. Further details NR.			100% SSD (clinical records). Further details NR.
			Physical	8					
			Threat	15					
Smith et al., 2018^70^	80	CFI^68^		Lifetime prevalence		Mixed relation sample. Further details NR.			100% Psychosis (clinical records). Further details NR.
			Property	5					
			Physical	14					

Abbreviations: BPAD, bipolar disorder; DSM‐IV, Diagnostic and Statistical Manual of Mental Disorders, Fourth Edition; Dx, diagnosis; ICD‐10, International Statistical Classification of Diseases 10th revision; IO, illness onset; MDD, major depressive disorder; NR, not reported; SSD, schizophrenia spectrum disorder; SZ, schizophrenia; SZA, schizoaffective disorder; VV, violent victimisation.

^a^ Table structured as: (a) studies reporting patient violence perpetration, and (b) studies reporting relative violent victimisation. Studies are ordered by time period assessed (i.e., shortest to longest), and alphabetised (by author).

^b^ Eligibility crtieria required some experience of violent victimisation; relatives reported victimisation rates for types of violence during month preceding the study.

### 3.3 Prevalence rates of violence

Prevalence of violence was reported as patient's self‐reported perpetration against relatives (*n* = 2), and relative's self‐reported victimisation (*n* = 25). Prevalence rates and further details are provided in Table 1.

#### 3.3.1 Patient‐reported family violence perpetration

Two studies reported an overall rate of family violence perpetration reported by patients, ranging from 19% over a 12‐month period,^28^ to 41% over a 2‐month period.^43^ Both studies reported on outpatient samples. The former study investigated patients' reports of physical violence causing injury and/or use of lethal weapons to threaten or harm relatives,^28^ while the latter employed a broader operationalisation encompassing multiple types of violence against parents.^43^ This study also reported perpetration rates disaggregated by violence type; physical and financial violence were most prevalent, followed by verbal and psychological violence.^43^

#### 3.3.2 Relative/carer‐reported violent victimisation

##### 3.3.2.1 Overall prevalence of violent victimisation

Three studies defined violence broadly to include multiple types, but did not disaggregate prevalence by type.^34^, ^40^, ^59^ Prevalence of victimisation ranged from 21% in the preceding 6 months,^40^ to 77% in the preceding 12 months.^59^ Both studies sampled relatives of community‐dwelling patients. Loughland and colleagues' operationalisation of violence included physical, verbal, and sexual types,^59^ while Labrum and Solomon investigated physical acts, and threats with a leathal weapon.^40^

##### 3.3.2.2 Prevalence of victimisation by type of violence

In two studies, inclusion criteria required that relatives had experienced patient violence to be eligible.^37^, ^51^ Therefore, these were not included in the assessment of victimisation prevalence, reported below. However, in both studies, relatives reported rates of different types of victimisation in the month preceding the study. Of types studied (i.e., verbal, physical, and property destruction), the reported prevalence of verbal violent victimisation was highest in both studies, compared with physical victimisation and property destruction.

##### Physical violence

In 21 studies reported prevalence of physical violent victimisation among sampled relatives.^4^, ^14^, ^20^, ^29^, ^33^, ^40^, ^42^, ^45^, ^49^, ^53^, ^55^, ^56^, ^57^, ^58^, ^63^, ^65^, ^66^, ^67^, ^69^, ^70^ This ranged from a lifetime prevalence of 8% reported by a sample of siblings,^69^ to 76% of sampled relatives who were of mixed relation to patients, but predominately mothers (comprising 84% of those sampled).^67^ Both studies sampled relatives of community‐dwelling patients. Definitions of physical violence in the sibling study were restricted to being struck or injured,^69^ while Kageyama and colleagues^67^ defined physical violence more broadly (e.g., punched, strangled, beaten with object).

##### Threats

Five studies reported the prevalence of threatened violence against relatives,^4^, ^33^, ^45^, ^49^, ^69^ ranging from lifetime prevalence rates of 15% of sampled siblings,^69^ to 58% of a mixed relative sample during the 2‐weeks preceding patients' last hospitalisation.^49^ One study reported 6‐month prevalence of threats involving a lethal weapon of 4%.^42^

##### Property destruction

In six studies, relatives reported the prevalence of patient property destruction,^4^, ^14^, ^33^, ^45^, ^49^, ^70^ ranging from lifetime prevalence rates of 5%,^70^ to 45% over a 2‐week period.^49^ Both studies reported on relatives of mixed relation to community‐dwelling patients. However, Lauber et al.^49^ considered the 2‐week period preceding patients' last hospitalisation.

##### Verbal violence

Four studies reported the prevalence of verbal violent victimisation in sampled relatives.^4^, ^29^, ^33^, ^45^ This ranged from a 12‐month prevalence of 42%, reported by sampled relatives of community‐dwelling patients,^45^ to 62% of relatives in the 12‐months preceding patients' hospitalisation.^4^

##### Psychological violence

Six studies reported the prevalence of psychological violent victimisation in sampled relatives.^20^, ^53^, ^55^, ^58^, ^65^, ^67^ Rates ranged from a lifetime prevalence of 56%,^65^ to 88%.^67^ Both studies used equivalent definitions of psychological violence, and predominantly sampled mothers who were mostly co‐resident with the patient.^65^, ^67^

##### Financial violence

In two studies, 6‐month prevalence rates of financial violent victimisation in sampled relatives, predominately mothers were 19% and 20%, respectively.^53^, ^55^ Both studies defined financial violence as the misuse or stealing of funds, property, or assets, and reported on relatives of mixed relation to community‐dwelling patients.

#### 3.3.3 Prevalence of violence by diagnosis

Of studies reporting on mixed diagnostic samples, two reported prevalence rates of violence disaggregated by patient diagnosis.^4^, ^33^ One study reported no significant differences in relatives' victimisation rates by patient diagnosis.^4^ The other reported that relatives of patients diagnosed with schizophrenia and BPAD, compared with MDD, reported significantly more violent victimisation.^33^ Three studies explored potential associations between patient diagnosis and violence. The authors reported no significant links between: perpetration and primary diagnosis of psychotic compared with affective disorder^28^; or victimisation in relatives and schizophrenia‐related or BPAD diagnoses.^40^, ^71^ Six studies did not comment on relationships between diagnosis and violence (see Table 1).

#### 3.3.4 Prevalence of violence by relationship type

Of studies reporting on samples of mixed family relationships (e.g., siblings, mothers, fathers, partners), six reported prevalence rates disaggregated by relationship type.^4^, ^34^, ^41^, ^43^, ^56^, ^63^ Parents, specifically mothers, were most likely to be reported victims of patient violence. However, in one study, victimisation was greatest in the adult sons of patients.^34^ Another found no significant differences in rates of victimisation by relationship type.^41^ Four studies reported examining associations between relationship type and violence^40^, ^53^, ^59^, ^66^; two found no significant associations.^59^, ^66^ Labrum^53^ sampled parents and spouses/partners of patients; adjusted odd's ratios indicated that psychological victimisation was significantly less likely in parents, but no significant differences in physical or financial violent victimisation were found by relationship type. Labrum and Solomon^40^ reported that being a parent was negatively associated with victimisation, after controlling for other significant covariates (e.g., greater caregiving hours).^40^ In 17 studies did not comment on violence by relationship type (see Table 1).

### 3.4 Impacts of patient violence on relatives

A total of 12 quantitative studies examined how violence impacted relatives.^4^, ^14^, ^33^, ^34^, ^37^, ^45^, ^58^, ^59^, ^65^, ^67^, ^69^, ^70^ Violent victimisation was significantly associated with negative impacts on relatives' mental health (e.g., psychological distress, PTSD symptoms), and the relative‐patient relationship (e.g., more negative emotions/attitudes towards the patient). Table 2 details the assessment of impacts and key findings reported by studies.

**TABLE 2:** Quantitative studies examining impacts of patient violence on relatives

Author, year	Impact assessed	Specific outcome assessed	Measure	Key findings
Hanzawa et al., 2013^34^	Psychological Relationship	PTSD Burden	Impact of Event Scale‐Revised (IESR)^71^; Zarit Caregiver Burden Interview^72^	Mean IESR scores were higher in relatives reporting VV vs. no VV (*t* = 2.82, *P* = 0.006). Mean burden scores were higher in relatives reporting VV vs. no VV (*t* = 3.924, *P* < 0.001).
Kageyama et al., 2016^58^	Psychological	Distress	K6^73^	VV was significantly greater in high‐distress group, for psychological VV (OR = 1.99, 95% CI 1.07–3.69) and physical VV (OR = 4.54, 95% CI 2.36–8.75).
Kageyama et al., 2018^67^	Psychological Relationship	Suicidality Emotions/ attitudes	Study‐specific questions	Relatives reporting only psychological VV vs. relatives reporting any physical VV reported thoughts of: suicide (*P* = 0.004); murder‐suicide (*P* = 0.002); and wished for the patient's death (*P* = 0.0002) significantly more often.
Kageyama & Solomon, 2018^65^	Psychological	PTSD	IESR^71^	IESR scores significantly higher in relatives reporting VV likely to result in severe injury (OR = 2.03, 95% CI 1.09–3.80; “never experienced” = reference).
Loughland et al., 2009^59^	Psychological	Distress PTSD	Depression Anxiety & Stress Scale^74^; IESR^71^	No significant differences in distress scores based on severity of VV (none‐mild vs. moderate–severe groups' scores were in normal range). High likelihood of PTSD in 52% of relatives reporting moderate–severe VV. Verbal VV (*F* (1, 64) = 7.29, *P* = 0.009), but not physical or sexual VV, was significantly higher in the high‐likelihood of PTSD subgroup (vs. low‐likelihood).
Onwumere et al., 2014^14^	Psychological Relationship	Distress Self‐esteem Burden Emotions/ attitudes	General Health Questionnaire (GHQ‐28)^75^; Rosenberg Self‐Esteem Scale^76^; Experience of Caregiving Inventory (ECI)^77^; Camberwell Family Interview (CFI)^68^	Physical VV was not significantly associated with distress or reported burden in relationship with patient (*P* > 0.05). Relatives reporting physical VV (vs. no VV) reported: greater hostility towards patients (*t* = 2.201, df = 68, *P* = 0.03); and lower self‐esteem (*t* = 2.199, df = 60, *P* = 0.03).
Smith et al., 2018^70^	Psychological Relationship	Mental wellbeing Burden Emotions/ attitudes	RAND 36‐item health survey^78^; ECI^77^; CFI^68^;	Relatives reporting VV (vs. no VV) reported: poorer mental wellbeing (*P* < 0.05, *d* = 0.664), more: negative caring experiences (*P* < 0.05, *d* = 0.547); hostility towards patient (*P* < 0.05, *d* = 0.644); criticism and EE towards the patient (*P* < 0.005, ϕc = 0.35).
Smith & Greenberg 2008^69^	Relationship	Quality	Positive Affect Index^79^	A worse quality of sibling relationship was associated with: threats of and physical VV (*β* = −0.18, *p* < 0.05); and greater fear of patient (*β* = −0.17, *p* < 0.05).
Vaddadi et al., 1997^33^	Psychological Relationship	Distress Burden	GHQ‐28^75^; Burden on Family Interview (BFI)^61^	VV was correlated with: distress (*r* = +0.31, *P* < 0.01); and burden in relationship with patient (*r* = +0.46, *P* < 0.001).
Vaddadi et al., 2002^45^	Psychological Relationship	Distress Burden	GHQ‐28^75^; BFI^61^	VV was correlated with distress (*r* = +0.35, *P* < 0.01) (scores indicated potential psychiatric morbidity in 60% of relatives); and burden in relationship with patient (*r* = +0.58, *P* < 0.01).
Varghese et al., 2016^37^	Relationship	Emotions/ attitudes Quality	Impact of Patient Aggression on Carers Scale (Adapted)^80^	Following VV: 91% of relatives reported adverse moral emotions towards, and 42% reported impaired relationship with, the patient.
Wang et al., 2019^4^	Physical Psychological Relationship	Injury Mental wellbeing Emotions/ attitudes	Study‐specific	Following VV, reported injuries were: 58% pain; 27% soft tissue; 16% trauma; 3% fracture; 3% cerebral concussion. Mental wellbeing complaints: 64% grievance; 58% anger; 41% helplessness; 40% low mood; 34% fear and insecurity; 1% suicidal thoughts; 22% reported feelings of hatred.

Abbreviation: VV, violent victimisation.

### 3.5 Qualitative synthesis

The qualitative synthesis sought to explore relatives' perceptions of their experiences of violence and associated impacts. Data were drawn from 11 qualitative studies,^30^, ^31^, ^32^, ^35^, ^36^, ^38^, ^39^, ^44^, ^81^, ^82^, ^83^ and one mixed methods.^29^ In all studies, definitions of violence included physical violence towards relatives. Studies also included: property destruction^83^; verbal^29^, ^35^; psychological^38^, ^39^, ^44^; financial^38^, ^39^, ^44^; sexual violence^44^; and threatening,^44^, ^82^ controlling and coercive behaviours.^44^ Violence was assessed over different time intervals comprising: participants' lifetime^32^, ^38^, ^39^, ^82^, ^83^; since patient illness onset^29^, ^81^; and 12 months preceding the study.^30^, ^31^ Three studies did not comment on time frame.^35^, ^36^, ^44^ Four themes were identified: (i) the nature of violence; (ii) physical impacts; (iii) psychological/emotional impacts; and (iv) impacts on familial relationships (see Table 3).

**TABLE 3:** Experiences and impacts of patient violence: Themes and quotes

Theme	Subtheme	Key supporting participant quotes (source; study reference)	Studies featuring theme (study references)
1. The nature of violence	1.1. Uncontrollability	*Accumulated anger and severe irritability suddenly exploded*. (Father: Hsu et al., 2014)^31^	^29^, ^30^, ^31^, ^32^, ^35^, ^36^, ^38^, ^44^, ^81^, ^82^, ^83^
	1.2. Escalation	*Most of the time it was screaming and throwing things, kicking the walls, breaking the doors, slamming the doors and throwing anything that's on the shelf off the shelves. Then it got to the point where it was starting to get physical*. (Mother: Copeland & Heilemann, 2008)^82^	^31^, ^32^, ^36^, ^39^, ^44^, ^81^, ^82^, ^83^
	1.3. Repetitiveness	*I was beaten 20 or 30 times by him, whose face was like a devil, and I was bloodied*. (Parent: Kageyama et al., 2018)^83^	^29^, ^30^, ^31^, ^36^, ^38^, ^39^, ^44^, ^81^, ^82^, ^83^
2. Physical impacts (injury)		*And then he sprayed me with the insect spray. I choked, I could not breathe… I have to be careful that he will not get hold of a knife*. (Mother; Band‐Winterstein et al., 2016)^38^ *Two or three ribs were cracked by kicking*. (Parent; Kageyama et al., 2018)^83^	^30^, ^32^, ^36^, ^38^, ^39^, ^83^
3. Psychological/emotional impacts	3.1. Trauma	*[Violence], it stays for life, afterward. Those images never go away*. (Daughter: Paradis‐Gange et al., 2020)^32^	^30^, ^32^, ^36^, ^83^
	3.2. Fear	*When I was younger, I could overcome him faster, save myself, now that I'm old and I have diabetes, now I have to be faster, and I got triglycerides in my blood. Now I'm afraid for my life, afraid he will kill me*. (Mother: Band‐Winterstein et al., 2014)^39^	^29^, ^30^, ^31^, ^32^, ^35^, ^36^, ^38^, ^39^, ^44^, ^81^, ^82^, ^83^
	3.3. Burden	*It's affected me physically, mentally, spiritually; every aspect of it has affected me* … *at times, most times, I dunno what to do… oh I'm getting tired of it now. (*Mother: Onwumere et al., 2019)^81^	^29^, ^30^, ^31^, ^32^, ^35^, ^36^, ^38^, ^39^, ^44^, ^81^, ^82^, ^83^
	3.4. Responsibility	*I do not want to live with him but I have no choice… he cannot live alone and I make sure he does not sleep out in the street… there is no‐one else to take care of him*. (Mother: Band‐Winterstein et al., 2014)^39^	^29^, ^30^, ^31^, ^32^, ^35^, ^36^, ^38^, ^39^, ^44^, ^81^, ^82^, ^83^
4. Impacts on familial relationships	4.1. Communication	*It affected it a lot because now we cannot even talk. We cannot be friends. Because he thinks I'm his enemy, so we cannot have a relationship. We do not talk too much because he gets annoyed*. (Mother: Onwumere et al., 2019)^81^ *I wanted my parents to understand my pain. However, I could not talk to them and damaged the walls at home instead*. (Patient: Kageyama et al., 2019)^44^	^30^, ^31^, ^32^, ^36^, ^38^, ^39^, ^44^, ^81^, ^83^
	4.2. Family‐level repercussions	*Your life is intense. You walk around on eggshells. Your life is not happy, especially when they get really, really ill… sometimes my whole family has this intensity of drama and anxiety*. (Mother: Copeland & Heilemann, 2008)^82^	^30^, ^31^, ^32^, ^36^, ^44^, ^82^, ^83^
	4.3. Family breakdown	*I could not trust him, and my home was not a safe place for either of us. When the social worker contacted me to have him released, I had to make some decisions to tell her that he could not come back to my home, which was, as any time I have to do something hard like that, it was heartbreaking. It was very hard*. (Mother: Sporer, 2019)^36^	^30^, ^31^, ^32^, ^36^, ^38^, ^39^, ^44^, ^81^, ^83^

#### 3.5.1 Theme 1: The nature of violence

##### Uncontrollability

Perceptions of patient violence as uncontrollable were captured in all studies. Relatives shared challenges in understanding how to prevent or control violent episodes, conveying a sense of powerlessness. Uncontrollability was also echoed in patients' narratives.^30^, ^44^

##### Escalation

Escalation in violence was common, and often discussed in terms of increased severity.^31^, ^32^, ^36^, ^82^ Escalation in the types of violence relatives experienced was also described; progressing from verbal, to property destruction, to physical violence.^82^ Often, relatives attributed patient violence to specific illness symptoms, such as delusions and hallucinations, and symptom deterioration.^32^, ^35^, ^81^, ^83^ Many studies described relatives' reluctance to seek help, until violence escalated and required intervention from external agencies, including the police.^32^, ^36^, ^39^, ^44^, ^81^, ^82^, ^83^

##### Repetitiveness

The repetitive nature of violence was captured in most studies.^29^, ^30^, ^31^, ^36^, ^38^, ^39^, ^44^, ^81^, ^82^, ^83^ For some, violence occurred on a daily basis, while for others violence had remained ongoing over several years.

#### 3.5.2 Theme 2: Physical impacts (injury)

Studies described the physical impact of violence in terms of physical injuries.^30^, ^32^, ^36^, ^38^, ^39^, ^83^ Disclosed injuries included: bruises^32^; cracked ribs^83^; back injury^39^; scars^30^; and choking and feeling unable to breathe.^38^

#### 3.5.3 Theme 3: Psychological/emotional impacts

##### Enduring psychological injury (trauma)

In contrast to immediate physical injuries, were enduring psychological injuries.^30^, ^32^, ^36^, ^83^ A daughter described how the mental images of violence, committed by a parent, had stayed with her for life.^32^ In another study, a mother described how suffering “trauma on a day‐to‐day basis” as a result of patient violence, had damaged her own, and her healthy daughter's, mental health.^36^ This was reflected in another study, where the “damage” to mental health extended to all relatives.^83^

##### Fear

Feeling fearful was described by participants in all studies, and drove efforts to seek help. Fear was most related to the potential for further violence, some described fearing for their lives. Many described a process of (hyper)vigilance whenever the patient was in close proximity; some avoided the patient entirely. Expressions of “walking on eggshells”,^81^, ^82^ waiting for something “horrible” to happen, and living in fear of what might happen were common.^35^, ^38^, ^39^, ^44^, ^82^, ^83^

##### Burden

Family burden was captured in most studies. Relatives described: enduring immense suffering attributed to years of repeated violence^31^, ^38^, ^39^; feeling physically, mentally and spiritually affected by violence^81^; feeling isolated and alone in shouldering the burden of violence^32^, ^38^, ^81^; and feeling exhausted.^31^, ^81^, ^83^ Burden fluctuations were also captured in relatives' accounts. For example, reductions in burden typically coincided with periods where patients were absent from the home^32^, ^35^, ^36^, ^38^, ^39^, ^81^, ^82^; mostly because of psychiatric hospitalisation. Relatives felt a sense of relief in knowing that the patient was safe in hospital.^35^, ^81^, ^82^ Hospitalisation provided respite for relatives but was accompanied by feelings of guilt because of their involvement in facilitating patients' hospitalisations, and the relief they felt. Respite and the freedom it offered was often bittersweet.^33^, ^37^

##### Responsibility

The concept of responsibility featured across all studies. Relatives frequently expressed feelings of self‐blame and responsibility for the patient's violence.^29^, ^35^, ^44^, ^81^, ^83^ Self‐blame attributions were common in parents' narratives; some communicated consequently tolerating violence. Responsibility was also linked to the ongoing role that relatives assumed in caring for the patient.^30^, ^31^, ^32^, ^35^, ^36^, ^38^, ^39^, ^81^, ^82^ This responsibility was often unwavering in the context of violence and prominent in parental accounts. Parental roles were perceived as “never ending”; caregiving was not a choice, but an accepted part of life.^38^

#### 3.5.4 Theme 4: Impacts on familial relationships

##### Communication

Patients and victimised relatives reported that family communications were negatively affected by violence and communication barriers perpetuated violence.^30^, ^31^, ^32^, ^36^, ^38^, ^39^, ^44^, ^81^, ^83^ Their accounts reflected a disconnect, where communication was both lacking and fraught. Patients attributed this disconnect to relatives' failure to understand their pain of living with SMI. The disconnect yielded feelings of frustration, alienation and being misunderstood, which preceded violence.^44^ Conversely, relatives expressed fear around communication and provoking further violence. Avoidance of potentially difficult conversations with the patient (including discussing violent incident(s)) and disturbing the status quo were highlighted.^30^, ^44^, ^81^, ^83^

##### Family‐level repercussions

In many accounts, discussions about patient violence illustrated its impact overall family. Home environments were described as being constantly tense and distressing by relatives^30^, ^31^, ^32^, ^36^, ^44^, ^82^, ^83^ and patients.^30^ Relatives also described the ways in which their relationships with other family members had deteriorated because of patient violence.

##### Family breakdown

Family fragmentation and separation were identified impacts of violence recurrence.^30^, ^31^, ^32^, ^36^, ^38^, ^39^, ^44^, ^81^, ^83^ Some described separation as a temporary precaution (e.g., escaping from the home).^30^, ^31^, ^44^, ^81^, ^83^ In other cases, relatives chose to live separately from the patient, because of safety issues.^36^, ^83^

## 4 DISCUSSION

This systematic review of 38 papers examined the types, rates, and impacts of violence by individuals diagnosed with SMI towards their relatives. Relative samples were mostly female, while males comprised a greater proportion of patient samples. Few studies commented on ethnic composition but, where reported, samples mostly comprised patients and relatives classified as white. Types and rates of patient violence against relatives and their impacts varied significantly between studies.

Variation in rates of reported violence might partly reflect methodological differences between studies, including operationalisations of violence.^84^ While some studies narrowly focused on physical violence, others included multiple types. Heterogeneity existed between studies with regards to: measurement of violence; time periods assessed; patient diagnostic groups; and familial relationship types. This may also have impacted variation in reported rates. Study heterogeneity precluded firm conclusions about which type of violence was most prevalent. However, findings from reviewed studies that operationalised violence more broadly and reported prevalence disaggregated by type, indicated a higher frequency of reports of psychological and verbal violent victimisation, than physical victimisation. Importantly, where violence was operationalised broadly, relatives frequently reported experiencing different types. This supports literature evidencing co‐occurrence of violence types,^19^ and cautions against the use of restrictive definitions when examining patient violence towards relatives. However, many reviewed studies did not disaggregate rates of violence by key variables (e.g., type of violence; patient diagnosis), so conclusions are tentative.

That said, the current findings concur with wider literature suggesting that mothers are the group most likely to be victims of patient violence, when compared with other relatives,^85^ and the general public.^86^ Caregiving is typically female dominated^18^; as the onset of SMI often occurs in early adulthood, patients may remain living with or receiving support from family of origin, particularly mothers.^87^ Previous literature asserts that accessibility,^88^ and increased contact,^85^, ^89^ may increase violence risk by individuals with SMI against relatives. Collectively, these findings offer partial explanation about elevated victimisation rates in mothers. Relative samples were predominantly female, and 13 studies exclusively sampled parents of patients. Accordingly, mothers comprised the largest proportion of all sampled relatives. Female relatives, particularly mothers, may be overrepresented in research as they are more likely to be the identified primary caregivers of patients, and research volunteers.^90^ This is an acknowledged bias within the literature, and is common when sampling participants from SMI family support groups.^41^, ^67^ Among the reviewed studies that included mixed relation samples and reported prevalence of violent victimisation disaggregated by relationship type, findings highlighted that other relatives (including, but not limited to siblings, children and spouses) also report experiencing patient violence,^4^, ^34^, ^63^ and negative sequalae^32^, ^35^, ^36^, underlining the importance of ensuring the inclusion of a broader range of relatives in carer research.

Most studies sampled relatives of patients diagnosed with schizophrenia spectrum disorders. These diagnostic groups are not homogenous. Furthermore, studies including mixed diagnostic samples rarely disaggregated rates of violence by patient diagnosis, examined for potential associations between violence and diagnosis, or accounted for potentially shared symptoms between diagnostic groups. Collectively, this creates challenges in determining whether the risk of family violence by people living with SMI is more strongly associated with any one diagnosis or is better conceptualised as a transdiagnostic risk. Researchers advocate that violence by people with SMI may be better understood by shifting focus from diagnostic categories to examining specific symptoms^89^, ^91^; the experience of which may be shared across various diagnostic groups. Key symptoms of schizophrenia‐related disorders (e.g., paranoid ideation) have been associated with specific forms of violence (e.g., general, but not IPV).^92^, ^93^ Further, family violence perpetration, compared with violence against non‐relatives, was elevated among individuals with SMI experiencing specific symptoms (i.e., persecutory delusions, command hallucinations).^94^

The findings from quantitative studies identified that violent victimisation was significantly associated with negative impacts to relatives' mental health, and familial relationships. The qualitative synthesis confirmed the detrimental impacts of violence on victimised relatives, patients, and wider family networks. Home environments were often characterised by reports of burden, and (hyper)vigilance. Reductions in burden were noted when the patient was absent from the home, offering families respite and relief. The value of respite care for SMI carers is under‐researched. However, benefits of respite, for carers and care recipients, are well‐documented within the wider literature.^95^

The qualitative findings speak to the important role of family communication. Fraught communications were described as a consequence of violence, and barriers to effective communication were felt to perpetuate violence. In patient qualitative accounts, violence was linked to frustrations of feeling misunderstood by relatives. This supports literature highlighting elevated risks of family violence perpetration in patients who report not feeling listened to,^89^ and who struggle to communicate their concerns.^96^ Additionally, our quantitative findings showed that relatives' communication styles characterised by criticism and hostility towards patients, correlated significantly with their violent victimisation.^14^, ^70^ This concurs with research suggesting that violence may arise in contexts when patients perceive criticism and hostility (i.e., high expressed emotion [EE]) within familial environments.^20^ Family interventions for psychosis, included in treatment guidelines, are effective in improving poor relationships characterised by high EE,^97^ supporting the value of targeting relationship quality when providing family‐based psychosocial interventions around violence prevention.^98^

Finally, the qualitative synthesis highlighted that relatives often felt responsible to assume the role of carer for patients. Relatives' commitment to caring remained prioritised, even in the context of violence.

### 4.1 Limitations

This review included only peer‐reviewed and English language published papers. However, mental health problems and domestic violence are recognised global public health crises in non‐English speaking nations.^99^, ^100^ Relatedly, many studies did not comment on ethnicity but, where reported, samples mostly comprised participants classified as white; overlooking the experiences of non‐white and/or racially minoritised groups who can have higher rates of SMI diagnoses.^101^

Quality appraisal identified several common methodological issues across the reviewed studies (e.g., recruitment and sample size). Collectively, these issues raise questions about selection and non‐response bias, sample representativeness, and transferability of findings beyond populations studied. All studies examined violence using self‐report measures, indicating a possibly high risk of reporting bias. Given that socially undesirable behaviours tend to be underreported, self‐report methods may additionally underestimate true prevalence.^92^ Indeed, patient violence is often underreported by relatives, because of fears surrounding the possible consequences of disclosure for those involved.^81^ Relatives were often sampled independently of patients. Studies, therefore, relied on relatives' reports to confirm the patient's diagnosis and consequently lacked independent confirmation of accuracy, which might have led to an over or under estimation of prevalence. Moreover, comorbidities (e.g., autism spectrum disorders), that themselves have been associated with violence,^102^ may have been overlooked.

There is substantial evidence to suggest that, when compared with the general population, the increased risk of violence perpetration in people with SMI is partly attributable to comorbid substance misuse.^103^ Further, irrespective of whether primary or comorbid diagnoses, substance use disorders carry the highest absolute and relative risks of domestic violence.^93^ Of the reviewed studies: some accounted for patients' comorbid substance use in analyses; some excluded patients with comorbid substance use, and others made limited/no reference to whether comorbid substance use had been examined or accounted for. This limits the ability to develop a more detailed understanding of the contexts in which family violence occurs and the potential role of substance misuse, specifically where violence by people with SMI is perpetrated against relatives. Finally, although the reviewed qualitative studies provided an insight into impacts of violent victimisation, examining impact was not the focus of most studies; restricting the amount of data interrogation that could be undertaken.

### 4.2 Implications

#### Clinical

Health professionals encounter challenges in identifying and responding to domestic violence.^104^ Ensuring identification of families exposed to patient violence therefore necessitates *explicit* enquiry. This approach serves as an important early step, given that patient violence towards relatives often goes unreported to legal authorities.^86^ The review underlines the importance of raising staff awareness about family violence, and their readiness and competency to respond to disclosures.^105^, ^106^ Our findings attesting the role of communication issues in facilitating violence also have implications for mental health and domestic violence services. Family interventions that aim to reduce risk factors for patient violence may benefit from greater emphasis on the promotion of effective communication and de‐escalation skills for relatives.^107^ Finally, this review highlighted that caregiving relationships do not necessarily cease following patient violence. Relatives, particularly primary caregivers (e.g., parents), may continue to provide care to patients, despite risk. This underscores the importance and underlying rationale for the development and implementation of psychosocial interventions, designed to reduce risks of patient violence in families and ongoing caregiving relationships.^81^

#### Research

Further research exploring family violence committed by people with SMI is warranted and should proceed with a broader, standardised operationalisation that encompasses the different types of violence.^23^, ^24^ Given the co‐occurrence of different types of violence, disaggregation of rates by type is recommended. To facilitate a more detailed understanding, future studies may additionally benefit from the inclusion of patients with shared symptom presentations (e.g., persecutory delusions) instead of generic SMI diagnoses. More research is required to understand the subjective experiences and impacts of patient violence on relatives, including consideration of how these may differ depending on key socio‐demographic attributes including ethnicity, gender, and relationship type. These insights are integral to exploring the varying support needs of families affected, and may help to inform the development of theory‐driven, tailored interventions that target specific needs.^97^ Future studies may benefit from employing qualitative and/or mixed methods to provide a more nuanced understanding of the lived experience.^17^, ^108^ Future research should also endeavour to cast a wider recruitment net, ensuring the inclusion of hitherto underrepresented relative groups (e.g., children, racial and ethnic minority groups). Relatedly, reviewed studies rarely sampled patients. Eliciting patients' perspectives may therefore facilitate the development of a more comprehensive understanding.^70^, ^81^

### 4.3 Conclusion

To conclude, not withstanding different definitions, descriptions and sensationalist media reports, violence by people living with SMI towards their relatives does happen. The negative impacts extend beyond the victimised relative and relative‐patient dyad, to the wider family network. Development of a framework that acknowledges the interdependency of family experiences and the wider consideration of the family as an integrated unit in future research and practice is indicated.^109^

## FUNDING INFORMATION

For Emilie K. Wildman, Juliana Onwumere, Deirdre MacManus, and Elizabeth Kuipers this paper represents independent research [part] funded by the National Institute for Health and Care Research (NIHR) Biomedical Research Centre at South London and Maudsley NHS Foundation Trust and King's College London. The views expressed are those of authors and not necessarily those of the NHS, the NIHR, King's College London or the Department of Health.

## CONFLICT OF INTEREST

The authors declare no conflicts of interest.

## TRANSPARENT PEER REVIEW

The authors did not opt in for transparent peer review for this article.

## Supporting information


**Appendix S1** Supporting Information

## Data Availability

Data sharing is not applicable to this article as no new data were created or analyzed in this study.

## Footnotes

* Papers were excluded if only aggregate age was reported (e.g., mean) and if this equalled <16.0.

† Studies focusing exclusively on these issues were beyond the scope of this review, which examined the prevalence of different types of patient‐perpetrated violence against relatives, and the associated impacts for victimised relatives.

‡ Two studies reported on the same parent sample (n = 16).^38^, ^39^ Three studies reported on the same relative sample (n = 573) of mixed relation to patients.^40^, ^41^, ^42^

## References

[1] International Alliance of Carer Organizations . Global State of Caring 2021. https://internationalcarers.org/global-state-of-care/

[2] American Psychiatric Association . Diagnostic and Statistical Manual of Mental Disorders: DSM‐5. 5th ed. American Psychiatric Publishing; 2013.

[3] Goldhagen RFS , Davidtz J . Violence, older adults, and serious mental illness. Aggress Violent Behav. 2020;57:1‐10.

[4] Wang L , Xu J , Zou H , Zhang H , Qu Y . Violence against primary caregivers of people with severe mental illness and their knowledge and attitudes towards violence: a cross‐sectional study in China. Arch Psychiatr Nurs. 2019;33(6):167‐176. 31753224 10.1016/j.apnu.2019.08.009

[5] Carers UK . Facts about carers 2019. 2019. https://www.carersuk.org/images/Facts_about_Carers_2019.pdf

[6] Kuipers E , Onwumere J , Bebbington P . Cognitive model of caregiving in psychosis. Br J Psychiatry. 2010;196(4):259‐265. 20357299 10.1192/bjp.bp.109.070466

[7] Onwumere J , Lotey G , Schulz J , et al. Burnout in early course psychosis caregivers: the role of illness beliefs and coping styles. Early Interv Psychiatry. 2017;11(3):237‐243. 25721376 10.1111/eip.12227

[8] Sin J , Sin J , Elkes J , et al. Mental health and caregiving experiences of family carers supporting people with psychosis. Epidemiol Psychiatr Sci. 2021;30(e3):1‐9. Accessed 25 March, 2021. doi:10.1017/S2045796020001067 PMC7116786 33416043

[9] Mittendorfer‐Rutz E , Rahman S , Tanskanen A , et al. Burden for parents of patients with schizophrenia‐a nationwide comparative study of parents of offspring with rheumatoid arthritis, multiple sclerosis, epilepsy, and healthy controls. Schizophr Bull. 2019;45(4):794‐803. 30184197 10.1093/schbul/sby130 PMC6581137

[10] Wasserman S , De Mamani AW , Suro G . Shame and guilt/self‐blame as predictors of expressed emotion in family members of patients with schizophrenia. Psychiatry Res. 2012;196(1):27‐31. 22357355 10.1016/j.psychres.2011.08.009 PMC3465708

[11] Gupta S , Isherwood G , Jones K , Van Impe K . Assessing health status in informal schizophrenia caregivers compared with health status in non‐caregivers and caregivers of other conditions. BMC Psychiatry. 2015;15(1):1‐11. 26194890 10.1186/s12888-015-0547-1 PMC4509463

[12] Hayes L , Hawthorne G , Farhall J , O'Hanlon B , Harvey C . Quality of life and social isolation among caregivers of adults with schizophrenia: policy and outcomes. Community Ment Health J. 2015;51(5):591‐597. 25690154 10.1007/s10597-015-9848-6

[13] Angermeyer MC , Bull N , Bernert S , Dietrich S , Kopf A . Burnout of caregivers: a comparison between partners of psychiatric patients and nurses. Arch Psychiatr Nurs. 2006;20(4):158‐165. 16846776 10.1016/j.apnu.2005.12.004

[14] Onwumere J , Grice S , Garety P , et al. Caregiver reports of patient‐initiated violence in psychosis. Can J Psychiatry. 2014;59(7):376‐384. 25007421 10.1177/070674371405900705 PMC4086313

[15] Hyde AP . Coping with the threatening, intimidating, violent behaviors of people with psychiatric disabilities living at home: guidelines for family caregivers. Psychiatr Rehabil J. 1997;21(2):144‐149. Accessed 17 September, 2020.

[16] Thompson MS . Violence and the costs of caring for a family member with severe mental illness. J Health Soc Behav. 2007;48(3):318‐333. 17982871 10.1177/002214650704800308

[17] Solomon PL , Cavanaugh MM , Gelles RJ . Family violence among adults with severe mental illness: a neglected area of research. Trauma Viol Abus. 2005;6(1):40‐54. Accessed 16 September, 2020. https://pubmed.ncbi.nlm.nih.gov/15574672/ 10.1177/1524838004272464 15574672

[18] Onwumere J , Zhou Z , Kuipers E . Informal caregiving relationships in psychosis: reviewing the impact of patient violence on caregivers. Front Psychol. 2018;9:1‐14. 30233448 10.3389/fpsyg.2018.01530 PMC6129604

[19] de Girolamo G , Bianconi G , Boero ME , et al. Studying patients with severe mental disorders who act violently: Italian and European Projects. In: Violence and Mental Disorders. Springer, Cham; 2020:155‐179.

[20] Chan B . Violence against caregivers by relatives with schizophrenia. Int J Forensic Ment Health. 2008;7(1):65‐81.

[21] Labrum T , Zingman MA , Nossel I , Dixon L . Violence by persons with serious mental illness toward family caregivers and other relatives: a review. Harv Rev Psychiatry. 2020;29(1):10‐19. 10.1097/HRP.0000000000000263 33417373

[22] Moher D , Liberati A , Tetzlaff J , et al. Preferred reporting items for systematic reviews and meta‐analyses: the PRISMA statement. Ann Intern Med. 2009;151(4):264‐269. 19622511 10.7326/0003-4819-151-4-200908180-00135

[23] United Nations (UN) . What is domestic abuse? 2019. https://www.un.org/en/coronavirus/what-is-domestic-abuse

[24] Home Office . Domestic Abuse: Statutory Guidance 2022. https://assets.publishing.service.gov.uk/government/uploads/system/uploads/attachment_data/file/1089015/Domestic_Abuse_Act_2021_Statutory_Guidance.pdf

[25] Thomas J , Harden A . Methods for the thematic synthesis of qualitative research in systematic reviews. BMC Med Res Methodol. 2008;8(1):1‐10. 18616818 10.1186/1471-2288-8-45 PMC2478656

[26] Hawker S , Payne S , Kerr C , Hardey M , Powell J . Appraising the evidence: reviewing disparate data systematically. Qual Health Res. 2002;12(9):1284‐1299. 12448672 10.1177/1049732302238251

[27] Downes MJ , Brennan ML , Williams HC , Dean RS . Development of a critical appraisal tool to assess the quality of cross‐sectional studies (AXIS). BMJ Open. 2016;6(12):e011458. 10.1136/bmjopen-2016-011458 PMC5168618 27932337

[28] Elbogen EB , Swanson JW , Swartz MS , Van Dorn R . Family representative payeeship and violence risk in severe mental illness. Law Hum Behav. 2005;29(5):563‐574. 16254743 10.1007/s10979-005-7120-2

[29] Ferriter M , Huband N . Experiences of parents with a son or daughter suffering from schizophrenia. J Psychiatr Ment Health Nurs. 2003;10(5):552‐560. 12956634 10.1046/j.1365-2850.2003.00624.x

[30] Hsu MC , Tu CH . Adult patients with schizophrenia using violence towards their parents: a phenomenological study of views and experiences of violence in parent‐child dyads. J Adv Nurs. 2014;70(2):336‐349. 23855926 10.1111/jan.12194

[31] Hsu MC , Huang CY , Tu CH . Violence and mood disorder: views and experiences of adult patients with mood disorders using violence toward their parents. Perspect Psychiatr Care. 2014;50(2):111‐121. 24689491 10.1111/ppc.12028

[32] Paradis‐Gagné E , Holmes D , Perron A . Experiences of family violence committed by relatives with severe mental illness: a grounded theory. J Forensic Nurs. 2020;16(2):108‐117. 31977516 10.1097/JFN.0000000000000272

[33] Vaddadi KS , Soosai E , Gilleard CJ , Adlard S . Mental illness, physical abuse and burden of care on relatives: a study of acute psychiatric admission patients. Acta Psychiatr Scand. 1997;95(4):313‐317. 9150825 10.1111/j.1600-0447.1997.tb09637.x

[34] Hanzawa S , Bae JK , Bae YJ , et al. Psychological impact on caregivers traumatized by the violent behavior of a family member with schizophrenia. Asian J Psychiatr. 2013;6(1):46‐51. 23380317 10.1016/j.ajp.2012.08.009

[35] Kontio R , Lantta T , Anttila M , Kauppi K , Välimäki M . Family involvement in managing violence of mental health patients. Perspect Psychiatr Care. 2015;53(1):55‐66. 26382565 10.1111/ppc.12137

[36] Sporer K . Aggressive children with mental illness: a conceptual model of family‐level outcomes. J Interpers Violence. 2019;34(3):447‐474. 27080308 10.1177/0886260516641283

[37] Varghese A , Khakha DC , Chadda RK . Pattern and type of aggressive behavior in patients with severe mental illness as perceived by the caregivers and the coping strategies used by them in a tertiary care hospital. Arch Psychiatr Nurs. 2016;30(1):62‐69. 26804503 10.1016/j.apnu.2015.10.002

[38] Band‐Winterstein T , Avieli H , Smeloy Y . Harmed? Harmful? Experiencing abusive adult children with mental disorder over the life course. J Interpers Violence. 2016;31(15):2598‐2621. 25854589 10.1177/0886260515579505

[39] Band‐Winterstein T , Smeloy Y , Avieli H . Shared reality of the abusive and the vulnerable: the experience of aging for parents living with abusive adult children coping with mental disorder. Int Psychogeriatrics. 2014;26(11):1917‐1927. 10.1017/S1041610214001495 25075607

[40] Labrum T , Solomon PL . Factors associated with family violence by persons with psychiatric disorders. Psychiatry Res. 2016;244:171‐178. 27479109 10.1016/j.psychres.2016.07.026

[41] Labrum T , Solomon PL . Rates of victimization of violence committed by relatives with psychiatric disorders. J Interpers Violence. 2017;32(19):2955‐2974. 26231334 10.1177/0886260515596335

[42] Labrum T , Solomon PL . Acts of weapon threat and use against family members by persons with psychiatric disorders. Violence Gend. 2016;3(2):89‐91.

[43] Fawzi MH , Fawzi MM , Fouad AA . Parent abuse by adolescents with first‐episode psychosis in Egypt. J Adolesc Health. 2013;53(6):730‐735. 23954728 10.1016/j.jadohealth.2013.07.004

[44] Kageyama M , Yokoyama K , Horiai Y . Perceptions of stages of family violence and their perceived solutions in persons with schizophrenia. Open Nurs J. 2019;13(1):156‐167.

[45] Vaddadi KS , Gilleard C , Fryer H . Abuse of carers by relatives with severe mental illness. Int J Soc Psychiatry. 2002;48(2):149‐155. 12182510 10.1177/002076402128783208

[46] Ghanizadeh A , Jafari P . Risk factors of abuse of parents by their ADHD children. Eur Child Adolesc Psychiatry. 2010;19(1):75‐81. 19820986 10.1007/s00787-009-0067-y

[47] Robins LN , Helzer JE , Croughan J , Ratcliff KS . National institute of mental health diagnostic interview schedule: its history, characteristics, and validity. Arch Gen Psychiatry. 1981;38(4):381‐389. 6260053 10.1001/archpsyc.1981.01780290015001

[48] Swanson JW , Holzer CE , Ganju VK , Jono RT . Violence and psychiatric disorder in the community: evidence from the epidemiologic catchment area surveys. Hosp Community Psychiatry. 1990;41(7):761‐770. 2142118 10.1176/ps.41.7.761

[49] Lauber C , Eichenberger A , Luginbühl P , Keller C , Rössler W . Determinants of burden in caregivers of patients with exacerbating schizophrenia. Eur Psychiatry. 2003;18(6):285‐289. 14611923 10.1016/j.eurpsy.2003.06.004

[50] Kluiter H , Kramer JJAM , Wiersma D . Interview for Measuring the Burden on the Family (IBF). Department of Social Psychiatry, University of Groningen; 1998.

[51] Madathumkovilakath NB , Kizhakkeppattu S , Thekekunnath S , Kazhungil F . Coping strategies of caregivers towards aggressive behaviors of persons with severe mental illness. Asian J Psychiatr. 2018;35:29‐33. 29751218 10.1016/j.ajp.2018.04.032

[52] Yudofsky SC , Silver JM , Jackson W , Endicott J , Williams D . The overt aggression scale for the objective rating of verbal and physical aggression. Am J Psychiatry. 1986;143(1):35‐39. 3942284 10.1176/ajp.143.1.35

[53] Labrum T . Factors related to abuse of older persons by relatives with psychiatric disorders. Arch Gerontol Geriatr. 2017;68:126‐134. 27810660 10.1016/j.archger.2016.09.007

[54] Monahan J , Steadman HJ , Silver E , et al. Rethinking Risk Assessment: the MacArthur Study of Mental Disorder and Violence. Oxford University Press; 2001.

[55] Labrum T , Solomon PL , Bressi SK . Physical, financial, and psychological abuse committed against older women by relatives with psychiatric disorders: extent of the problem. J Elder Abus Negl. 2015;27(4–5):377‐391. 10.1080/08946566.2015.1092902 26371747

[56] Kageyama M , Solomon P , Kita S , et al. Factors related to physical violence experienced by parents of persons with schizophrenia in Japan. Psychiatry Res. 2016;243:439‐445. 27450747 10.1016/j.psychres.2016.06.036

[57] Kageyama M , Solomon P . Characteristics of parents with high expressed emotion and related factors: a study of parents of adults with schizophrenia. J Nerv Ment Dis. 2018;206(12):955‐961. 30439779 10.1097/NMD.0000000000000902

[58] Kageyama M , Solomon P , Yokoyama K . Psychological distress and violence towards parents of patients with schizophrenia. Arch Psychiatr Nurs. 2016;30(5):614‐619. 27654247 10.1016/j.apnu.2016.02.003

[59] Loughland CM , Lawrence G , Allen J , et al. Aggression and trauma experiences among carer‐relatives of people with psychosis. Soc Psychiatry Psychiatr Epidemiol. 2009;44(12):1031‐1040. 19333531 10.1007/s00127-009-0025-5

[60] Oud NE . Testing the perception of prevalence of aggression scale (POPAS). Paper presented at: The 2nd European Congress on Violence in Clinical Psychiatry (Stockholm; Amsterdam). 2001.

[61] Pai S , Kapur RL . The burden on the family of a psychiatric patient: development of an interview schedule. Br J Psychiatry. 1981;138(4):332‐334. 7272637 10.1192/bjp.138.4.332

[62] Straus MA , Hamby SL , Boney‐McCoy S , Sugarman DB . The revised conflict tactics scales (CTS2) development and preliminary psychometric data. J Fam Issues. 1996;17(3):283‐316.

[63] Kageyama M , Yokoyama K , Nagata S , et al. Rate of family violence among patients with schizophrenia in Japan. Asia‐Pacific J Public Heal. 2015;27(6):652‐660. Accessed 4 November, 2020. doi:10.1177/1010539515595069 26182940

[64] Kaplan HI, Sadock BJ, eds. Comprehensive Textbook of Psychiatry. Vols 1–2. 5th ed. Williams & Wilkins Co; 1989.

[65] Kageyama M , Solomon P . Post‐traumatic stress disorder in parents of patients with schizophrenia following familial violence. PLoS One. 2018;13(6):e0198164. 29856788 10.1371/journal.pone.0198164 PMC5983498

[66] Kageyama M , Solomon P . Physical violence experienced and witnessed by siblings of persons with schizophrenia in Japan. Int J Forensic Ment Health. 2019;48(1):2‐13.

[67] Kageyama M , Solomon P , Yokoyama K , Nakamura Y , Kobayashi S , Fujii C . Violence towards family caregivers by their relative with schizophrenia in Japan. Psychiatry Q. 2018;89(2):329‐340. 10.1007/s11126-017-9537-4 28971267

[68] Vaughn C , Leff J . The measurement of expressed emotion in the families of psychiatric patients. Br J Soc Clin Psychol. 1976;15(2):157‐165. 938822 10.1111/j.2044-8260.1976.tb00021.x

[69] Smith MJ , Greenberg JS . Factors contributing to the quality of sibling relationships for adults with schizophrenia. Psychiatr Serv. 2008;59(1):57‐62. 18182540 10.1176/appi.ps.59.1.57 PMC2396577

[70] Smith LM , Onwumere J , Craig T , Kuipers E . Caregiver correlates of patient‐initiated violence in early psychosis. Psychiatry Res. 2018;270:412‐417. 30308465 10.1016/j.psychres.2018.09.011

[71] Weiss DS , Marmar CR . The impact of event scale—revised. Assessing Psychological Trauma and PTSD. The Guilford Press; 1997:399‐411.

[72] Zarit SH , Zarit JM . The Memory and Behavior Problems Checklist. 1987R and the burdeninterview (technical report). University Park (PA): Pennsylvania State University; 1987.

[73] Kessler RC , Andrews G , Colpe LJ , et al. Short screening scales to monitor population prevalences and trends in non‐specific psychological distress. Psychol Med. 2002;32(6):959‐976. 12214795 10.1017/s0033291702006074

[74] Lovibond SH , Lovibond PF . Manual for the Depression Anxiety Stress Scales. Vol 56; 1995.

[75] Goldberg D , Williams PA . A Users' Guide to the General Health Questionnaire. NFER‐Nelson. Windsor (GB); 1988.

[76] Rosenberg M . Society and the Adolescent Self‐Image. Princeton University Press; 1965.

[77] Szmukler GI , Burgess P , Herrman H , Benson A , Colusa S , Bloch S . Caring for relatives with serious mental illness: the development of the experience of caregiving inventory. Soc Psychiatry Psychiatr Epidemiol. 1996;31(3–4):137‐148. 8766459 10.1007/BF00785760

[78] Ware JE , Sherbourne CD . The MOS 36‐item short‐form health survey (Sf‐36): I. conceptual framework and item selection. Med Care. 1992;30(6):473‐483. 1593914

[79] Bengtson VL , Schrader SS . Parent‐child relations. In Handbook of Research Instruments in Social Gerontology, Vol 2. (Mangen D, Peterson W, eds.). University of Minnesota Press; 1982.

[80] Needham I , Abderhalden C , Halfens RJG , Dassen T , Haug HJ , Fischer JE . The impact of patient aggression on carers scale: instrument derivation and psychometric testing. Scand J Caring Sci. 2005;19(3):296‐300. 16101859 10.1111/j.1471-6712.2005.00344.x

[81] Onwumere J , Parkyn G , Learmonth S , Kuipers E . The last taboo: the experience of violence in first‐episode psychosis caregiving relationships. Psychol Psychother Theory Res Pract. 2019;92(1):1‐19. Accessed 4 November 2020. doi:10.1111/papt.12173 29399952

[82] Copeland DA , Heilemann MSV . Getting “to the point”: the experience of mothers getting assistance for their adult children who are violent and mentally ill. Nurs Res. 2008;57(3):1‐17. 18496098 10.1097/01.NNR.0000319500.90240.d3 PMC4107905

[83] Kageyama M , Yokoyama K , Nakamura Y , Kobayashi S , Fujii C . The coping process of Japanese parents who experience violence from adult children with schizophrenia. Arch Psychiatr Nurs. 2018;32(4):549‐554. 30029746 10.1016/j.apnu.2018.03.004

[84] Nederlof AF , Muris P , Hovens JE . The epidemiology of violent behavior in patients with a psychotic disorder: a systematic review of studies since 1980. Aggress Violent Behav. 2013;18(1):183‐189.

[85] Estroff SE , Swanson JW , Lachicotte WS , Swartz M , Bolduc M . Risk reconsidered: targets of violence in the social networks of people with serious psychiatric disorders. Soc Psychiatry Psychiatr Epidemiol. 1998;33(Suppl 1):S95‐S101. 9857786 10.1007/s001270050216

[86] Nordström A , Kullgren G . Do violent offenders with schizophrenia who attack family members differ from those with other victims? Int J Forensic Ment Health. 2003;2(2):195‐200.

[87] Jansen JE , Gleeson J , Cotton S . Towards a better understanding of caregiver distress in early psychosis: a systematic review of the psychological factors involved. Clin Psychol Rev. 2015;35:56‐66. 25531423 10.1016/j.cpr.2014.12.002

[88] Binder RL , McNiel DE . Victims and families of violent psychiatric patients. J Am Acad Psychiatry Law Online. 1986;14(2):131‐139. 3730626

[89] Swanson JW , Swartz MS , Van Dorn RA , et al. A National Study of violent behavior in persons with schizophrenia. Arch Gen Psychiatry. 2006;63(5):490. Accessed September 16, 2020. https://pubmed.ncbi.nlm.nih.gov/16651506/ 16651506 10.1001/archpsyc.63.5.490

[90] National Alliance for Caregiving . ON PINS & NEEDLES Caregivers of Adults with Mental Illness; 2016. Accessed 4 February, 2021. https://www.caregiving.org/wp‐content/uploads/2020/05/NAC_Mental_Illness_Study_2016_FINAL_WEB.pdf

[91] Fresán A , Apiquian R , De La Fuente‐Sandoval C , et al. Violent behavior in schizophrenic patients: relationship with clinical symptoms. Aggress Behav. 2005;31(6):511‐520. Accessed 16 September, 2020. doi:10.1002/ab.20060

[92] Coid JW , Ullrich S , Bebbington P , Fazel S , Keers R . Paranoid ideation and violence: meta‐analysis of individual subject data of 7 population surveys. Schizophr Bull. 2016;42(4):907‐915. Accessed 15 September, 2020. https://pubmed.ncbi.nlm.nih.gov/26884548/ 26884548 10.1093/schbul/sbw006 PMC4903063

[93] Yu R , Nevado‐Holgado AJ , Molero Y , et al. Mental disorders and intimate partner violence perpetrated by men towards women: a Swedish population‐based longitudinal study. PLOS Med. 2019;16(12):e1002995. Accessed 15 September, 2020. doi:10.1371/journal.pmed.1002995 31846461 PMC6917212

[94] Joyal CC , Côté G , Meloche J , Hodgins S . Severe mental illness and aggressive behavior: on the importance of considering subgroups. Int J Forensic Ment Health. 2011;10(2):107‐117.

[95] Vandepitte S , Van Den Noortgate N , Putman K , Verhaeghe S , Verdonck C , Annemans L . Effectiveness of respite care in supporting informal caregivers of persons with dementia: a systematic review. Int J Geriatr Psychiatry. 2016;31(12):1277‐1288. 27245986 10.1002/gps.4504

[96] Brucato G , Appelbaum PS , Lieberman JA , et al. A longitudinal study of violent behavior in a psychosis‐risk cohort. Neuropsychopharmacology. 2017;43(2):264‐271. Accessed 26 March, 2021. www.neuropsychopharmacology.org 28745307 10.1038/npp.2017.151 PMC5729561

[97] Claxton M , Onwumere J , Fornells‐Ambrojo M . Do family interventions improve outcomes in early psychosis? A systematic review and meta‐analysis. Front Psychol. 2017;8:1‐17. Accessed 29 March, 2021. 28396643 10.3389/fpsyg.2017.00371 PMC5366348

[98] Wibawa IR , Hamid AYS , Daulima NHC . A phenomenological study: family experience in expressed emotion in providing care for client with risk of aggressive behavior. Walailak J Sci Technol. 2020;17(5):450‐459. Accessed 26 March, 2021. https://wjst.wu.ac.th/index.php/wjst/article/view/4885

[99] Vigo D , Thornicroft G , Atun R . Estimating the true global burden of mental illness. Lancet Psychiatry. 2016;3(2):171‐178. 26851330 10.1016/S2215-0366(15)00505-2

[100] World Health Organization . Violence Against Women Prevalence Estimates, 2018. WHO; 2021 https://www.who.int/publications/i/item/violence‐against‐women‐prevalence‐estimates

[101] Rodrigues R , MacDougall AG , Zou G , et al. Risk of involuntary admission among first‐generation ethnic minority groups with early psychosis: a retrospective cohort study using health administrative data. Epidemiol Psychiatr Sci. 2020;29:e59 https://www.cambridge.org/core/product/identifier/S2045796019000556/type/journal_article 10.1017/S2045796019000556 PMC8061249 31610825

[102] van Buitenen N , Meijers J , van den Berg CJW , Harte JM . Risk factors of violent offending in mentally ill prisoners with autism spectrum disorders. J Psychiatr Res. 2021;143:183‐188. 34500347 10.1016/j.jpsychires.2021.09.010

[103] Whiting D , Fazel S . Epidemiology and risk factors for violence in people with mental disorders. In: Carpiniello B , Vita A , Menacci C , eds. Violence and Mental Disorders. Comprehensive Approach to Psychiatry. Springer; 2020.

[104] Ruijne RE , Kamperman AM , Trevillion K , et al. Assessing the acceptability, feasibility and sustainability of an intervention to increase detection of domestic violence and abuse in patients suffering from severe mental illness: a qualitative study. Front Psych. 2020;11:1‐10. doi:10.3389/fpsyt.2020.581031 PMC7655979 33192725

[105] Kalra N , Hooker L , Reisenhofer S , Di Tanna GL , García‐Moreno C . Training healthcare providers to respond to intimate partner violence against women. Cochrane Database Syst Rev. 2021;2021(5):1‐102. 10.1002/14651858.CD012423.pub2 PMC8166264 34057734

[106] World Health Organization . Caring for Women Subjected to Violence: A WHO Curriculum for Training Health‐Care Providers. WHO; 2019 http://www.who.int/reproductivehealth/publications/caring-for-women-subject-to-violence/en/

[107] Katz J , Medoff D , Fang J , Dixon LB . The relationship between the perceived risk of harm by a family member with mental illness and the family experience. Community Ment Health J. 2015;51(7):790‐799. 25535047 10.1007/s10597-014-9799-3 PMC4478288

[108] Yin M , Li Z , Zhou C . Experience of stigma among family members of people with severe mental illness: a qualitative systematic review. Int J Ment Health Nurs. 2020;29(2):141‐160. Accessed 16 September, 2020. https://pubmed.ncbi.nlm.nih.gov/31648408/ 31648408 10.1111/inm.12668

[109] Tiyyagura G , Bloemen EM , Berger R , et al. Seeing the Forest in family violence research: moving to a family‐centered approach. Acad Pediatr. 2020;20(6):746‐752. Accessed November 4, 2020. https://pubmed.ncbi.nlm.nih.gov/31991169/ 31991169 10.1016/j.acap.2020.01.010 PMC7381357

