# Supplementary material for: Prevalence of violence by people living with severe mental illness against their relatives and its associated impacts: A systematic review
Source: Acta Psychiatr Scand. 2023 Jan 8;147(2):155–74. doi: 10.1111/acps.13516 (PMC10107449; doi:10.1111/acps.13516)
Supplement: Supplementary file 1 — Appendix S1 Supporting Information [file ACPS-147-155-s001.docx]

**Systematic Review Search Strategy (Ovid Databases)**

1. *Domestic violence/
2. *Family conflict/
3. (emotional abuse OR emotional violence OR emotional aggression OR psychological abuse OR psychological violence OR psychological aggression OR controlling behaviour* OR coercive control OR verbal abuse OR verbal aggression OR verbal assault* OR verbal threat* OR threat* OR physical abuse OR physical violence OR physical aggression OR physical assault* OR physical injur* OR abus* OR batter OR battered OR beaten OR beat OR attack* OR aggress* OR injur* OR sexual abuse OR sexual violence OR sexual aggression OR sexual assault* OR rape* OR domestic abuse OR domestic violence OR homicide* OR family violence OR intimate partner violence OR partner violence OR partner abuse OR spousal abuse OR spouse abuse) ADJ3 (perpetrat* OR family OR families OR household OR domestic OR partner* OR intimate partner* OR marital OR spouse OR wife OR wives OR husband* OR boyfriend* OR girlfriend* OR brother* OR sister* OR sibling* OR father* OR mother* OR parent* OR son* OR daughter* OR child* OR carer* OR caregiver* OR informal care*).ab,ti.
4. *mental disorders/
5. Exp *psychotic disorders/
6. Exp *schizophrenia/
7. Exp *depressive disorder/
8. Exp *bipolar disorder/
9. *eating disorders/
10. *personality disorders/
11. severe mental illness* OR mental illness*.mp
12. paranoi* OR delusion* OR hallucinati* OR persecutory delusion*.mp
13. social anxiety disorder* OR social phobia* OR panic disorder* OR generalized anxiety disorder* OR generalised anxiety disorder* OR GAD OR anxiety disorder* OR posttraumatic stress disorder* OR PTSD OR acute stress disorder* OR obsessive-compulsive disorder* OR OCD.mp
14. 1 OR 2 OR 3
15. 4 OR 5 OR 6 OR 7 OR 8 OR 9 OR 10 OR 11 OR 12 OR 13
16. 14 AND 15

***Table 1*** Summary of Included Studies and Key Demographics of Samples

| **Author, Year** | **Country** | **Design**  **(clinical setting)** | **Sample studied** | **Relative relationship to patient** | **Relative demographics**  **N (% Female); Mean age (SD)/Range; Ethnicity** | **Patient demographics**  **N (% Female); Mean age (SD)/Range; Ethnicity; Diagnosis** |
| --- | --- | --- | --- | --- | --- | --- |
| **Mixed Methods Studies (N=1)** | | | | | | |
| Ferriter & Huband, 2003^29^ | UK | Cross-sectional  (inpatient) | Family | 100% Parents | N=26 (62% F); Mean age = 60.8; Range=41-79; 100% White | N=22 (9% F); Mean age = 34.6; Range = 22-48; 100% White; 100% SZ |
| **Quantitative Studies (N=26)** | | | | | | |
| Kageyama & Solomon, 2018^57^ | Japan | Cross-sectional  (community) | Family | 100% Parents | N=289 (74% F); Mean age = 69 (SD 7.6); Ethnicity NR | N=283 (37% F); Mean age = 39.1 (SD 7.9); Ethnicity NR; 100% SZ |
| Kageyama & Solomon, 2018^65^ | Japan | Cross-sectional (community) | Family | 100% Parents | N=353 (68% F); Mean age = 69 (SD 7.3); Ethnicity NR | N =346 (37% F); Mean age = 38.8 (SD 7.7); Ethnicity NR; 100% SZ |
| Kageyama et al., 2016^56^ | Japan | Cross-sectional (community) | Family | 100% Parents | N=400 (67% F); Mean age = 69.2 (SD 7.5); Ethnicity NR | N=392 (37% F); Mean age = 39 (SD NR); Ethnicity NR; 100% SZ |
| Kageyama et al., 2016^58^ | Japan | Cross-sectional (community) | Family | 100% Parents | N=379 (68% F); Mean age = 69 (SD 7.7); Ethnicity NR | N=372 (36% F); Mean age = 38.8 (SD NR); Ethnicity NR; 100% SZ |
| Kageyama & Solomon, 2019^66^ | Japan | Cross-sectional (community) | Family | 100% Siblings | N=113 (57% F); Mean age = 41.5 (SD 11.7); Ethnicity NR | N=113 (37% F); Mean age = 40.9 (SD 10.9); Ethnicity NR; 100% SZ |
| Smith & Greenberg, 2008^69^ | USA | Cross-sectional (community) | Family | 100% Siblings | N=136 (55% F); Mean age = 44.5 (SD 8.7); 95% White; 3% African American; 2% Hispanic | N=136 (% NR); Mean age/range = NR; Ethnicity NR; 100% SSD |
| Chan, 2008^20^ | Canada | Cross-sectional  (community) | Family | 44% Mothers 18% Fathers 12% Spouses 8% Sisters  7% Brothers | N=61 (61% F); Mean age = 51.64 (SD 14.1); Ethnicity NR | N=51 (31% F); Mean age = 36.4 (SD 12.69); Ethnicity NR; 100% SZ |
| Hanzawa et al., 2013^34^ | Korea | Cross-sectional (inpatient & community) | Family | 54% Parents  25% Siblings  9% Children  12% NR | N=116 (55% F); Mean age = 55.3 (SD 13.5); Ethnicity NR | N=116 (33% F); Mean age = 40 (SD 10); Ethnicity NR; 100% SZ |
| Kageyama et al., 2018^67^ | Japan | Cross-sectional (community) | Family | 84% Mothers 12% Fathers  1% Spouses  2% Sisters | N=277 (% NR); Mean age = 68.6 (SD 7.7); Ethnicity NR | N=272 (36% F); Mean age = 39.9 (SD 8.9); Ethnicity NR; 100% SZ |
| Kageyama et al., 2015^63^ | Japan | Cross-sectional (community) | Family | 95% Parents  5% NR | N=301 (83% F); Mean age = 68.7 (SD 7.9); Ethnicity NR | N=302 (38% F); Mean age = 40.1 (SD 8.9); Ethnicity NR; 100% SZ |
| Labrum, 2017^53^ | USA | Cross-sectional  (community) | Family | 77% Parents  11% Spouses  12% NR | N=243 (89% F); Mean age = 62 (SD 6.18); 94% White | N=243 (36% F); Mean age = 38.3 (SD 14.41); 91% White; 42% BPAD; 38% SSD; 9% MDD; 7% Anxiety- related; 4% NR |
| ^a^ Labrum & Solomon, 2016^40^, 2016^42^, 2017^41^ | USA | Cross-sectional (community) | Family | 47% Parents  20% Spouses  15% Siblings  12% Children  7% NR | N=573 (87% F); Mean age = 48.94 (SD 14.63); 88% White; 4% Hispanic; 4% African American; 1% Asian; 0.5% American Indian | N=573 (39% F) Mean age=39.3 (SD 15.2); 85% White; 6% Hispanic; 5% American Indian; 2% Asian; 0.9% American Indian; 40% BPAD; 31% SSD; 13% MDD; 10% Anxiety; 5% Other (Depression, PTSD, ADD) |
| Labrum et al., 2015^55^ | USA | Cross-sectional (community) | Family | 80% Mothers  8% Spouses  6% Sisters  1% Daughters  5% Other | N=217 (100% F); Mean age = 62 (SD 5.95); 94% White; 2% Hispanic; 2% African American; 0.5% Asian; 0.5% American Indian | N=217 (34% F); Mean age=37.4 (SD 13.94); 91% White; 3% Hispanic; 2% African American; 2% Asian; 0.5% American Indian; 38% SSD; 42% BPAD; 8% MDD; 6% Anxiety-related; 4% Other (Non-Major Depression, PTSD, ADD) |
| Lauber et al., 2003^49^ | Switzerland | Cross-sectional (community) | Family | 77% Parents  23% NR | N=64 (58% F); Mean age = 61 (SD NR); Ethnicity NR | N=64 (33% F) Mean age = 39 (SD NR); Ethnicity NR; 100% SZ |
| Loughland et al., 2009^59^ | Australia | Cross-sectional (community) | Family | 49% Parents  32% Siblings  19% Children | N=106 (84% F); Mean age = 54.6 (SD 13.6); Ethnicity NR | N=106 (42% F); Mean age = 42.7 (SD 16.6); Ethnicity NR; 100% SSD |
| Madathumkovilakath et al., 2018^51^ | India | Cross-sectional (community) | Family | 49% Parents 35% Spouses 8% Siblings  7% Children | N=270 (76% F); Mean age= NR; Range=20-80; Ethnicity NR | N=270 (% NR); Mean age/range= NR; Ethnicity NR; 64% MD; 36% SSD |
| Smith et al., 2018^70^ | UK | Cross-sectional (community) | Family | 85% Mothers  5% Fathers  10% NR | N=80 (86% F); Mean age = 51.9 (SD 8.8); 35% White; 13% Black; 16% Black Caribbean; 11% Black African | N=80 (31% F); Mean age = 24.6 (SD 4.7); 35% White; 13% Black; 16% Black Caribbean; 11% Black African; 100% Psychosis |
| Varghese et al., 2016^37^ | India | Cross-sectional (inpatient & community) | Family | 48% Parents  29% Spouses  14% Siblings  9% Children | N=100 (56% F); Mean age = 40.7 (SD 13.2); Ethnicity NR | N=100 (38% F); Mean age = 31.8 (SD 9.23); Ethnicity NR: 48% SZ; 45% BPAD; 7% Psychosis |
| Wang et al., 2019^4^ | China | Cross-sectional (inpatient) | Family | 55% Parents  20% Spouses  15% Siblings  8% Children | N=208 (55% F) Mean age = 47.5 (SD 12.8); Ethnicity NR | N=208 (38% F) Mean age = 34.9 (SD 13.6); Ethnicity NR; 58% SZ; 30% BPAD; 12% SZA |
| Fawzi et al., 2013^43^ | Egypt | Cross-sectional (outpatient) | Patient | 100% Parents | Demographics NR | N=150 (45% F); Mean age = 16.6 (SD 1.8); Ethnicity NR; 100% Psychosis |
| Elbogen et al., 2005^28^ | USA | Cohort  (outpatient) | Patient | NR | Demographics NR | N=245 (45% F); Mean age = 40.3 (SD 10.7); 67% African American; 33% White; 72% SSD; 28% BPAD/MDD |
| Onwumere et al., 2014^14^ | UK | Cross-sectional (inpatient & community) | Both | 55% Parents 35% Partners 9% Siblings 1% Children | N=72 (72% F); Mean age = 52.9 (SD 12.9); 90% White; 4% Black | N=72 (27% F) Mean age = 35.8 (SD 12); 86% White; 6% Black; 100% Psychosis |
| Vaddadi et al., 2002^45^ | Australia | Cross-sectional (community) | Both | 66% Mothers  13% Spouses 9% Children  8% Fathers  4% NR | N=101 (73% F); Mean age = 56.9 (SD 13.9); Ethnicity NR | N=101 (36% F); Mean age = 36.3 (SD 12.2); Ethnicity NR; 64% SZ; 11% BPAD; 7% MDD; 18% NR |
| Vaddadi et al., 1997^33^ | Australia | Cross-sectional (inpatient) | Both | 47% Mothers  7% Fathers  32% Spouses 15% NR | N=101 (% F = NR); Mean age = 47.3 (SD 13.6); Ethnicity NR | N=100 (40% F); Mean age = 32.4  (SD 9.3); Ethnicity NR; 39% SZ; 27% MDD; 18% BPAD; 16% NR |
| **Qualitative studies (N=11)** | | | | | | |
| Copeland & Heilemann, 2008^83^ | USA | Community | Family | 100% Mothers | N=8 (100% F); Mean age = NR; Range = 42-60; 25% White; 25% African American; 12.5% African; 37.5% Latina/Hispanic | N=9 (33% F); Mean age = NR; Range = 20-38; Ethnicity NR; 100% Psychosis |
| ^b^ Band-Winterstein et al., 2014^39^, 2016^38^ | Israel | Community | Family | 100% Parents | N=16 (69% F); Mean age = NR; Range = 58-90; Ethnicity NR | N=16 (% NR); Mean age/range = NR; Ethnicity NR; 100% SZ |
| Kageyama, Yokoyama, et al., 2018^84^ | Japan | Community | Family | 100% Parents | N=26 (69% F); Mean age = 70.8; Range = 50-83; Ethnicity NR | N=24 (33% F); Mean age = 39.6; Range = 20-50; Ethnicity NR;  100% SZ |
| Kontio et al., 2015^35^ | Finland | Inpatient & Community | Family | Parents  Children  Spouses  (% NR) | N=8 (75% F); Mean age/ range = NR; Ethnicity NR | N=8 (% NR); Mean age/range = NR; Ethnicity NR; 100% SSD |
| Onwumere et al., 2019^82^ | UK | Community | Family | 75% Mothers  12.5% Fathers  12.5% Grandmothers | N=8 (88% F); Mean age = 56.6 (SD NR); 87.5% Black | N=8 (38% F); Mean age = 26.3 (SD NR); Ethnicity NR; 100% Psychosis |
| Paradis-Gagné et al., 2020^32^ | Canada | Inpatient | Family | 36% Mothers 29% Fathers 7% Sisters  7% Daughters  7% Other | N=14 (64% F); Mean age/ range = NR; Ethnicity NR | N=10 (% NR); Mean age = NR  Range = 25-65; Ethnicity NR; 80% SSD; 10% CD; 10% ASD |
| Sporer, 2019^36^ | USA | Inpatient & Community | Family | 40% Mothers  30% Fathers  20% Sisters  10% Brothers | N=10 (60% F); Mean age/ range = NR; 100% White | N=4 (25% F); Mean age = 16.5  Range = 8-21; Ethnicity NR; 75% BPAD; 25% Psychosis; 25% CD |
| Kageyama et al., 2019^44^ | Japan | Community | Both | 100% Mothers | N=5 (100% F); Mean age=NR; Range = 60-70; Ethnicity NR | N=5 (0% F); Mean age=NR; Range = 30-50; Ethnicity NR; 100% SZ |
| Hsu et al., 2014^31^ | Taiwan | Inpatient | Both | 100% Parents | N=13 (69% F); Mean age = 65.8 (SD 8.35); Ethnicity NR | N=13 (62% F); Mean age =36 (SD 6.2); Ethnicity NR; 100% MD |
| Hsu & Tu, 2014^30^ | Taiwan | Inpatient | Both | 100% Parents | N=14 (57% F); Mean age/ range = NR; Ethnicity NR | N=14 (29% F); Mean age = 35.6 (SD NR); Ethnicity NR; 100% SZ |
| *Note.* ^a-b^ Studies report on the same sample. Therefore, these are only reported once. *Abbreviations*. ADD = attention deficit disorder; ASD = autism spectrum disorder; BPAD = bipolar disorder; CD = conduct disorder; MDD = major depressive disorder; MD = mood disorder; NOS = not otherwise specified; NR = not reported; PTSD = post-traumatic stress disorder; SSD = schizophrenia spectrum disorder; SZ = schizophrenia; SZA = schizoaffective disorder. | | | | | | |

**Quality Appraisal of Quantitative Studies** (AXIS Tool, Downes et al., 2016)

| \| **Key:** \| \| \| \| --- \| --- \| --- \| \| Y \| \| Yes \| \| P \| \| Partial \| \| N \| \| No \| \| / \| \| N/A \| \| ? \| \| Unable to tell \| \|  \| \|  \| \|  \|  \| \| \|  \|  \| \| | Chan, 2008^20^ | ***** Elbogen et al., 2005^28^ | Fawzi et al., 2013^43^ | Hanzawa et al., 2013^34^ | Kageyama & Solomon, 2018^57^ | Kageyama & Solomon, 2018^65^ | Kageyama & Solomon, 2019^66^ | Kageyama et al., 2016^56^ | Kageyama et al., 2016^58^ | Kageyama et al., 2018^67^ | Kageyama et al., 2015^63^ | Labrum, 2017^53^ | Labrum & Solomon, 2016^42^ | Labrum & Solomon, 2016^40^ | Labrum & Solomon, 2017^41^ | Labrum et al., 2015^55^ | Lauber et al., 2003^49^ | Loughland et al., 2009^59^ | Madathumkovilakath et al., 2018^51^ | Onwumere et al., 2014^14^ | Smith et al., 2018^70^ | Smith & Greenberg, 2008^69^ | Vaddadi et al., 2002^45^ | Vaddadi et al., 1997^33^ | Varghese et al., 2016^37^ | Wang et al., 2019^4^ |
| --- | --- | --- | --- | --- | --- | --- | --- | --- | --- | --- | --- | --- | --- | --- | --- | --- | --- | --- | --- | --- | --- | --- | --- | --- | --- | --- | --- | --- | --- | --- | --- | --- | --- | --- | --- | --- | --- | --- | --- | --- | --- | --- | --- | --- | --- | --- | --- | --- | --- | --- | --- | --- | --- |
| ***TOTAL (%)*** | **68** | **78** | **83** | **65** | **73** | **68** | **70** | **78** | **70** | **68** | **63** | **85** | **78** | **85** | **93** | **84** | **63** | **83** | **70** | **85** | **75** | **68** | **63** | **63** | **68** | **86** |
| ***Introduction*** | | | | | | | | | | | | | | | | | | | | | | | | | | |
| Aims/objectives of study clear? | Y | Y | Y | Y | Y | Y | Y | Y | Y | Y | Y | Y | Y | Y | Y | Y | Y | Y | Y | Y | Y | Y | Y | Y | Y | Y |
| ***Methods*** | | | | | | | | | | | | | | | | | | | | | | | | | | |
| Study design appropriate for the stated aims? | Y | Y | Y | Y | Y | Y | Y | Y | Y | Y | Y | Y | Y | Y | Y | Y | Y | Y | Y | Y | Y | Y | Y | Y | Y | Y |
| Sample size justified? | N | N | N | N | N | N | N | N | N | N | N | N | N | N | N | N | N | N | N | N | N | N | N | N | N | N |
| Target population clearly defined? | Y | Y | Y | Y | Y | Y | Y | Y | Y | Y | Y | Y | Y | Y | Y | Y | Y | Y | Y | Y | Y | Y | Y | Y | Y | Y |
| Source of study population appropriate so that it closely represents target population? | Y | Y | Y | P | Y | Y | P | Y | Y | Y | Y | Y | Y | Y | Y | Y | Y | Y | P | Y | Y | Y | P | Y | P | P |
| Selection process likely to select participants that were representative of target population? | P | P | P | P | P | P | P | P | P | P | P | Y | Y | Y | Y | Y | P | ? | P | P | P | P | P | P | P | P |
| Measures taken to address and categorise non responders? | N | N | P | N | N | N | N | N | N | N | N | Y | Y | Y | Y | Y | N | Y | N | Y | N | N | P | P | N | / |
| Risk factors and outcome variables measured appropriate for study aims? | Y | Y | Y | Y | Y | Y | Y | Y | Y | Y | ? | Y | Y | Y | Y | Y | Y | Y | Y | Y | Y | Y | Y | Y | Y | Y |
| Variables measured correctly using instruments piloted or published previously? | Y | P | Y | Y | Y | P | Y | Y | P | N | N | Y | Y | Y | Y | P | Y | Y | Y | Y | Y | Y | Y | Y | Y | Y |
| Clear how statistical significance and/or precision estimates were determined? | P | Y | Y | Y | Y | P | P | Y | P | P | P | Y | / | Y | Y | / | P | Y | Y | Y | Y | P | P | P | Y | Y |
| Methods (including stats) sufficiently described to enable repetition? | Y | Y | Y | Y | Y | Y | Y | Y | Y | Y | Y | Y | P | Y | Y | Y | Y | Y | P | Y | Y | P | P | P | Y | Y |
| ***Results*** | | | | | | | | | | | | | | | | | | | | | | | | | | |
| Basic data adequately described? | Y | Y | Y | Y | Y | Y | Y | Y | Y | Y | Y | Y | Y | Y | Y | Y | Y | Y | Y | Y | Y | Y | P | Y | Y | Y |
| Response rate does not raise concerns about non-response bias? | N | P | P | ? | N | N | ? | P | N | N | N | Y | Y | Y | Y | Y | ? | Y | ? | N | N | N | N | P | ? | Y |
| If appropriate, information about non-responders described? | N | P | P | N | N | N | N | N | N | N | N | Y | Y | Y | Y | Y | N | Y | N | Y | N | N | P | N | N | / |
| Results internally consistent? | Y | Y | Y | P | P | P | Y | Y | Y | Y | Y | Y | Y | Y | Y | Y | Y | Y | Y | Y | Y | Y | Y | Y | Y | Y |
| Results presented for all analyses described in methods? | Y | Y | Y | Y | Y | Y | Y | Y | Y | Y | Y | Y | Y | Y | Y | Y | Y | Y | Y | Y | Y | P | P | P | Y | Y |
| ***Discussion*** | | | | | | | | | | | | | | | | | | | | | | | | | | |
| Authors’ discussions and conclusions justified by results? | P | P | P | P | P | P | P | P | P | P | P | P | N | P | P | P | P | P | P | P | P | P | P | P | P | P |
| Limitations discussed? | Y | Y | Y | N | Y | Y | Y | Y | Y | Y | Y | P | N | P | Y | Y | Y | Y | Y | Y | Y | Y | N | N | N | Y |
| ***Other*** | | | | | | | | | | | | | | | | | | | | | | | | | | |
| Includes funding/conflicts of interest statement that would not affect the authors interpretation of results? | N | P | Y | Y | Y | Y | Y | Y | Y | Y | Y | N | Y | N | Y | N | N | N | Y | Y | Y | Y | Y | N | Y | Y |
| Includes information to confirm ethical approval or consent attained? | Y | Y | Y | Y | Y | Y | Y | Y | Y | Y | Y | Y | Y | Y | Y | Y | N | Y | Y | Y | Y | Y | Y | Y | Y | Y |
| *Note.* Scoring: items answered as ‘yes’ received one point; a half point was awarded for items that were partially fulfilled; for items answered as ‘unable to tell’ no points were awarded; and for items that were not applicable, the total possible score was reduced by one increment. Total scores were converted to percentages to enable comparisons across studies. *****Elbogen et al., 2005 employed a cohort study design. | | | | | | | | | | | | | | | | | | | | | | | | | | |

**Quality Appraisal of Qualitative and Mixed Methods studies** (Hawker Tool, Hawker et al., 2002).

| \| **Key:** \| \| \| --- \| --- \| \| 1 \| Very poor \| \| 2 \| Poor \| \| 3 \| Fair \| \| 4 \| Good \| \|  \|  \| | Bandwinterstein et al., 2016^38^ | Bandwinterstein et al., 2014^39^ | Copeland & Heilemann, 2008^83^ | Ferriter and Hubband, 2003^29^ | Hsu et al., 2014^31^ | Hsu & Tu, 2014^30^ | Kageyama et al., 2019^44^ | Kageyama et al., 2018^84^ | Kontio et al., 2015^35^ | Onwumere et al., 2019^82^ | Paradis-Gange et al., 2020^32^ | Sporer, 2019^36^ |
| --- | --- | --- | --- | --- | --- | --- | --- | --- | --- | --- | --- | --- | --- | --- | --- | --- | --- | --- | --- | --- | --- | --- | --- | --- |
| Abstract and title | 3 | 3 | 4 | 3 | 3 | 4 | 3 | 2 | 3 | 4 | 3 | 3 |
| Introduction and aims | 3 | 3 | 3 | 3 | 3 | 4 | 3 | 3 | 3 | 3 | 3 | 3 |
| Method and data | 3 | 3 | 3 | 3 | 3 | 3 | 3 | 3 | 3 | 3 | 3 | 3 |
| Sampling | 3 | 3 | 2 | 2 | 3 | 3 | 3 | 2 | 2 | 2 | 3 | 4 |
| Data analysis | 3 | 3 | 3 | 3 | 4 | 4 | 3 | 4 | 3 | 3 | 3 | 3 |
| Ethics and bias | 3 | 3 | 3 | 3 | 4 | 4 | 3 | 2 | 2 | 2 | 3 | 1 |
| Findings/results | 3 | 3 | 4 | 3 | 3 | 4 | 3 | 4 | 3 | 4 | 3 | 3 |
| Transferability/generalisability | 3 | 3 | 2 | 2 | 3 | 3 | 3 | 2 | 2 | 2 | 3 | 3 |
| Implications and usefulness | 4 | 4 | 2 | 3 | 4 | 3 | 4 | 3 | 4 | 4 | 3 | 4 |
| **TOTAL** | **28**  **B** | **28**  **B** | **26**  **B** | **25**  **B** | **30**  **A** | **32**  **A** | **28**  **B** | **25**  **B** | **25**  **B** | **27**  **B** | **27**  **B** | **27**  **B** |
| *Note.* Overall quality score: calculated by assigning 1 point (very poor) to 4 points (good) for each answer. Scores range from minimum 9 points, to maximum 36 points. Overall quality grades: high quality (A), 30–36; medium quality (B), 24–29; low quality (C), 9–23. | | | | | | | | | | | | |
